# Understanding the Role of Self-Assembly and Interaction with Biological Membranes of Short Cationic Lipopeptides in the Effective Design of New Antibiotics

**DOI:** 10.3390/antibiotics11111491

**Published:** 2022-10-27

**Authors:** Oktawian Stachurski, Damian Neubauer, Aleksandra Walewska, Emilia Iłowska, Marta Bauer, Sylwia Bartoszewska, Karol Sikora, Aleksandra Hać, Dariusz Wyrzykowski, Adam Prahl, Wojciech Kamysz, Emilia Sikorska

**Affiliations:** 1 Faculty of Chemistry, University of Gdansk, Wita Stwosza 63, 80-308 Gdansk, Poland; 2 Faculty of Pharmacy, Medicinal University of Gdansk, Al. Gen. J. Hallera 107, 80-416 Gdansk, Poland; 3 Faculty of Biology, University of Gdansk, Wita Stwosza 59, 80-308 Gdansk, Poland

**Keywords:** antimicrobial peptides, lipopeptides, peptide–membrane interactions, self–assembly

## Abstract

This study investigates short cationic antimicrobial lipopeptides composed of 2–4 amino acid residues and C_12_-C_18_ fatty acids attached to the N-terminal part of the peptides. The findings were discussed in the context of the relationship among biological activity, self-assembly, stability, and membrane interactions. All the lipopeptides showed the ability to self-assemble in PBS solution. In most cases, the critical aggregation concentration (CAC) much surpassed the minimal inhibitory concentration (MIC) values, suggesting that monomers are the main active form of lipopeptides. The introduction of β-alanine into the peptide sequence resulted in a compound with a high propensity to fibrillate, which increased the peptide stability and activity against *S. epidermidis* and *C. albicans* and reduced the cytotoxicity against human keratinocytes. The results of our study indicated that the target of action of lipopeptides is the bacterial membrane. Interestingly, the type of peptide counterion may affect the degree of penetration of the lipid bilayer. In addition, the binding of the lipopeptide to the membrane of Gram-negative bacteria may lead to the release of calcium ions necessary for stabilization of the lipopolysaccharide layer.

## 1. Introduction

The invention of penicillin by Fleming was a ground-breaking discovery that heralded the era of antibiotics and changed the face of medicine. However, Fleming himself warned that the use of the drug sooner or later might lead to the emergence of drug-resistant bacteria [1]. Nowadays, drug resistance has impaired the effectiveness of almost all the antibiotics produced since the discovery of penicillin. Infections that no longer respond to antibiotics contribute to extended hospitalisation, higher treatment costs, and increased mortality. Scientists predict that by the year 2050, antibiotic resistance will kill more than 10 million people a year and the cost to the global economy may rise to USD 100 trillion [2,3]. Antimicrobial resistance is a natural phenomenon in which bacteria evolve to withstand the effects of drugs. However, the overuse of antibiotics together with non-compliance with treatment instructions accelerates the process. Moreover, the limited number of new drugs under development that could replace the ineffective ones also does not inspire optimism. To address the problem of antibiotic resistance, people need both behavioural changes and new strategies for fighting pathogens. The former is an individual matter, but it is important to raise awareness of drug resistance and to promote appropriate behaviour. In turn, the latter is a great challenge faced by modern science. Antimicrobial peptides (AMPs), produced by all living organisms [4,5,6], offer a promising alternative to conventional antibiotics. The bacteria produce AMPs to kill other bacteria competing for the same space. In the case of higher microorganisms, AMPs constitute important components of innate immunity, protecting the host against infections. These are relatively small compounds with a mass of up to 10 kDa and amphipathic structure. The presence of basic amino acid residues (Arg, Lys and His) provides a positive net charge and facilitates interactions with negatively charged membranes of pathogens [7]. Antimicrobial peptides have been reported as effective agents against both the Gram-positive and Gram-negative strains of bacteria, fungi, viruses, and parasites and also show immunomodulatory properties. The main mechanism of action of antimicrobial peptides is related to their ability to disrupt the biological membranes of pathogens. However, many AMPs can translocate across the membrane into the cytoplasm, where they target key cellular processes such as macromolecule synthesis, cell-division enzymatic activity, or autolysis [8,9]. By far the greatest advantages of antimicrobial peptides over conventional antibiotics are their biodegradability and relatively low propensity for the development of resistance. On the other hand, the high cost of synthesis, limited stability, short plasma half-life, low oral bioavailability, and unknown toxicology and pharmacokinetics are major barriers to AMPs in clinical use [10,11]. To overcome the obstacles to the clinical application of AMPs, several improvement strategies have been developed, including truncated sequences, the use of delivery systems, and chemical modifications. Conjugation of a peptide with a fatty acid deserves special attention among the latter. Lipidation may endow peptides with enhanced antimicrobial activities [12,13,14,15,16,17,18,19,20,21,22,23,24] and increase the enzymatic stability, bioavailability, and drug delivery potential [25,26,27]. Additional motivations to incorporate lipids into the peptide sequence are native antimicrobial lipopeptides such as daptomycin and polymyxins, produced nonribosomally by *Streptomyces roseosporus* and *Bacillus polymyxa*, respectively, or caspofungin, a semisynthetic derivative of pneumocandin Bo produced from a fermentation product of the fungus *Glarea lozoyensis*, all approved by the Food and Drug Administration (FDA) to clinical use [28,29].

**Figure 1:**
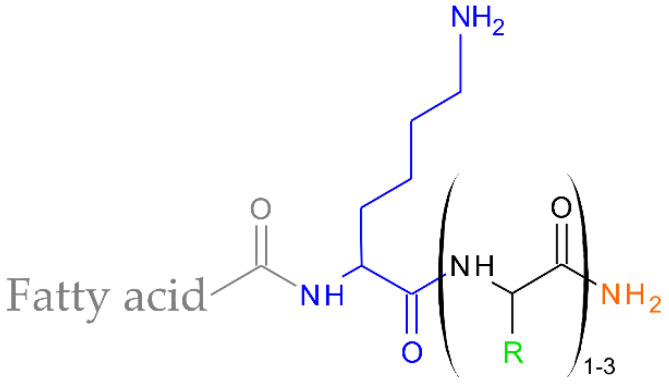
General structure of lipopeptides.

This study focused on ultra-short cationic lipopeptides consisting of 2–4 amino acid residues and C_12_–C_18_ fatty acids attached to the N-terminal part of the peptides (Figure 1). Short cationic lipopeptides seem to be an interesting alternative to naturally occurring AMP. They meet the conditions of amphipathicity and a cationic character, i.e., characteristics of almost all natural antimicrobial peptides. They are active against both planktonic forms and biofilms [30,31,32,33]. Moreover, their surfactant-like structure results in the ability to self-assemble in solution, a feature that might define selectivity [34]. The aim of this study was to determine the influence of the length of the fatty acid chain as well as the composition of the peptide sequence on antimicrobial and cytotoxic activities and the tendency to self-assembly. Moreover, the binding behaviour of the lipopeptides to the negatively charged as well as zwitterionic membranes has been examined. Ultimately, the findings were discussed in the context of a relationship among self-assembly, membrane interactions, and biological activity.

## 2. Results and Discussion

### 2.1. Antimicrobial Activity and Cytotoxicity

Antimicrobial assays showed that C_16_-KKKK-NH_2_ displays the broadest spectrum of activity over the concentration range of 8–16 µg/mL (Table 1). As is seen, truncation of the fatty acid tail reduced the overall activity, with the exception of activity against *S. epidermidis*, which remained still high. Moreover, the activity against *S. epidermidis* was improved after a single lysine to arginine substitution, which is compatible with our previous results on the lysine- and arginine-based lipopeptides [35,36]. The shortening of the fatty-acid tail as well as extending of the peptide chain by the addition of subsequent basic residues suppressed the hydrophobicity of the compounds and their antifungal activity. Accordingly, compounds with a hydrocarbon chain shorter than the palmitic one were not active against *C. albicans*. On the other hand, a decrease in the lipopeptide’s hydrophobicity reduced the toxicity against human red blood cells as measured by haemolytic activity (Figure S1, Supplementary Material), as well as to the human keratinocytes cell line (Table 1). Thus, C_16_-KKKK-NH_2_ seems to have an optimal balance of hydrophobic and polar fragments, which resulted in a suitable potency and relatively safe difference between the concentration of the lipopeptide that induced antimicrobial and cytotoxic activities.

### 2.2. Evaluation of Bacterial Cell Viability

Fluorophores SYTO 9 and propidium iodide (PI) were applied to assess the viability of *S. epidermidis* and *E. coli* bacterial cells treated with C_16_-KKKK-NH_2_, the compound with the broadest spectrum of antimicrobial activity among the lipopeptides studied. Both dyes bind to nucleic acids, but the former passes through both intact and damaged cell membranes, while the latter one penetrates only into cells with damaged membranes, so it is an excellent indicator of membrane integrity. All observations were carried out by fluorescence microscopy. As shown in Figures S2 and S3 (Supplementary Material), cells incubated with C_16_-KKKK-NH_2_ turned red to show that the lipopeptide damages the membrane of both *S. epidermidis* and *E. coli*. The effect was distinct even at the lowest peptide concentration used (1 × MIC). The results were compared with positive and negative controls, where the bacterial cells were treated either with 70% ethanol or cultured without any treatment, respectively. Ethanol disrupted the cell membranes, to stain the bacterial cells red, as was the case with the presence of the lipopeptide. In contrast, bacterial cells cultured without the lipopeptide still showed a green fluorescence, indicating undamaged membranes.

### 2.3. Stability in Serum

The stability in serum was performed for the selected lipopeptides. The first approach to the study showed that in UPLC (ultra-performance liquid chromatography) analysis, the retention times of the lipopeptides and the serum were comparable. Using either a mixture of acetonitrile, formic acid and deionized water or absolute ethanol as a “quenching solution” was insufficient to precipitate most of the proteolytic enzymes. Hence, the peptides were seen either as a peak shoulder of serum or were invisible on the RP-UPLC profile. Using 3% TCA (trichloroacetic acid) to quench the reaction and precipitate serum was satisfied because improved the RP-UPLC profiles of the degraded peptides. The serum stability assay demonstrated that two peptides with four Lys residues in the sequence C_14_-KKKK-NH_2_ and C_16_-KKKK-NH_2_ were digested faster than a single arginine substituted lysine counterparts after the first 30 min of incubation with serum. These lipopeptides were degraded up to 45% after 4 h, whereas those with arginine in the sequence were more stable and degraded up to 32–40% (Figure S1, Supplementary Material). The C_16_-KβAK-NH_2_ turned out to be distinctly more stable in the tests that the remaining peptides and even after 4 h of incubation with serum, it was degraded in only about 15%.

### 2.4. Self-Assembly

The critical aggregation concentrations (CACs) were determined in a phosphate buffer (pH 7.4) by surface tension measurements vs. the lipopeptide concentration (Table 2). A comparison of the CAC values obtained in the buffer with those previously determined in unbuffered solution [37] indicates an at least 10-fold decrease in CAC with an increase in the ionic strength of the solution. The phenomenon is more pronounced for peptides with a greater positive charge. An increase in ionic strength following the transition from the unbuffered to buffered solution facilities aggregation at a lower concentration due to more effective screening of the headgroup repulsion and tight packing molecules in a single aggregate.

As expected, lengthening the peptide chain led to an increase in the CAC value, which is related to the intensification of electrostatic repulsion between the peptide units and/or increase in the size of the headgroup. In turn, the CAC values decreased with elongation of the hydrocarbon chain length due to strengthening of the hydrophobic effect in accordance with a linear dependence between the logarithm of CAC and the number of carbons in the hydrocarbon chain for the compounds with the identical peptide entities (Figure S1, Supplementary Material). Interestingly, a comparison of this relationship for the Lys-based lipopeptides and previous Arg-based lipopeptides [38] showed that the CAC values decreased less drastically with the elongation of the hydrocarbon chain length for the former. Thus, the lauric-acid-modified tetra-L-lysine showed 0.9-fold lower CAC in PBS than its Arg-based counterpart, while with the palmitic-acid-conjugated derivatives, it was already four-fold higher. In addition, the self-diffusion coefficients extracted from the PFG NMR measurements for different lipopeptide concentrations (Figure S1, Supplementary Material) showed that C_16_-RRRR-NH_2_ self-assembled into distinctly larger aggregates than did C_16_-KKKK-NH_2_. These differences result from the different chemical characteristics of both amino acids. The guanidine group in the Arg side chain has a fairly unique ability to participate in hydrogen bonds and even favourable interactions with other guanidinium groups. Moreover, Arg can also sample conformational space more extensively than Lys, thus enhancing the probability of placing the guanidinium group near polar peptide atoms or bulky water, which stabilizes close arginine pairing. All those events are expected to facilitate the self-assembly [39,40,41]. In turn, the effect of a single substitution of lysine by arginine residue on the CAC value seems to depend on the position of the substitution. As shown in Table 2, the introduction of the arginine close to the surface of the self-assembled structure decreased the CAC only slightly, probably owing to its higher possibility to form hydrogen bonds with water molecules and increased counterion binding as compared to lysine, which reduced electrostatic repulsion. Again, Arg placed closer to the hydrophobic core led to an increase in CAC. According to the current knowledge, the Arg side chain retains its charge even when buried in a relatively hydrophobic interior of a protein. This is in contrast to Lys, whose side chain is readily deprotonated in the nonpolar microenvironment of proteins. We cannot also ignore the situation wherein deprotonation of some internal lysines takes place during the self-assembly process due either to desolvation or electrostatic repulsion between neighbouring lysine residues. In turn, the substitution of Lys with Arg residue shows a strong propensity to carry even a partially buried positive charge, which could strengthen electrostatic repulsion between peptide entities as well as increase the CAC value.

**Figure 2:**
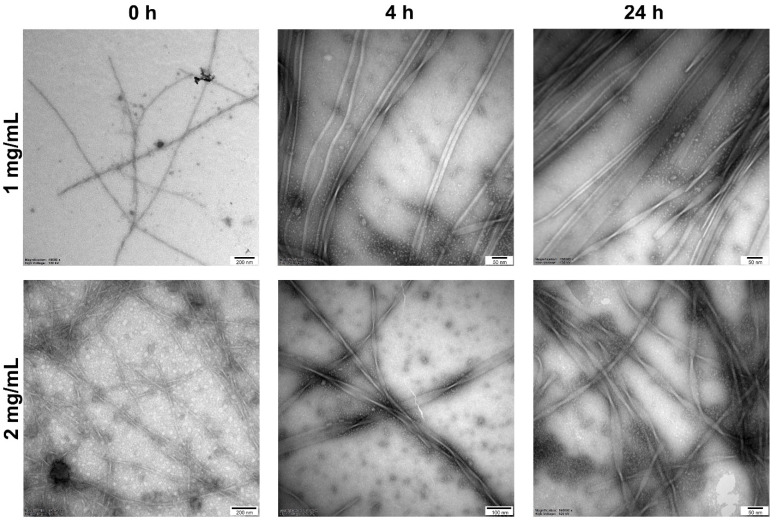
TEM images for the C_16_-KβAK-NH_2_ samples before (after dissolution), after 4 and 24 h of incubation of the peptide at concentrations of 1 mg/mL and 2 mg/mL.

A comparison of the results obtained for C_16_-KGK-NH_2_ and C_16_-KβAK-NH_2_ illustrated how simple modification of the structure can drastically affect the behaviour of the lipopeptide in solution. With regard to Gly, βAla is characterised by an additional methylene group between the carboxyl and amine functions, and this is enough for the peptide to form a precipitate at a low concentration and a gel-like structure at a higher one. This phenomenon made it impossible to determine the CAC value for C_16_-KβAK-NH_2_. However, as proved by the circular dichroism spectra recorded for C_16_-KβAK-NH_2_ in unbuffered and buffered solutions, the peptide appeared to display an unordered conformation with a minimum at 195 nm in the former, whilst it showed a spectrum characteristic of a β-sheet structure with a minimum around 219 nm in the latter (Figure S1, Supplementary Material). This result is compatible with the formation of a fibrose structure in the PBS solution [42,43]. In contrast, the conformation of the C_16_-KGK-NH_2_ remained unchanged, although the concentration of the peptide in PBS surpassed its CAC value. The thioflavin T-test was used for further studies of fibril formation. Thioflavin T (ThT) forms a complex with fibrils that generates a characteristic emission spectrum at 482 nm (Figure S1, Supplementary Materials). The C_16_-KβAK-NH_2_ showed a significant increase in the ThT emission intensity directly after dissolution. Then, over two hours of incubation, the ThT fluorescence intensity decreased down to a level of the blank and the peptide gradually precipitated out of the solution. However, further incubation initially led to a slow and then sharp re-increase in the ThT emission intensity, which indicates that the cyclic process occurred. This means that the fibrils were formed very quickly and precipitated out of the solution, which initiated the formation of other fibrils. For a deeper insight into the morphology of C_16_-KβAK-NH_2_ at different stages of incubation, transmission electron microscopy was employed (Figure 2). At a lower concentration (1 mg/mL), the peptide initially formed short and sparse fibrils, but with the extension of the incubation time, the fibrils became longer and longer (*ca.* 10–11 nm) and showed a tendency to twist. An increase in the initial concentration of the peptide (2 mg/mL) accelerated the fibrillation process and distinctly more fibrils were formed immediately upon dissolution. Further incubation led to the formation of curled (ribbon-like) fibrils of about 16 nm in diameter.

### 2.5. Isothermal Titration Calorimetry to Study Lipopeptide-Lipid Interaction

To confirm direct interactions between the lipopeptides and membrane lipids, isothermal titration calorimetry (ITC) was employed. In these experiments, the lipopeptides were titrated with individual lipids, POPC (1-palmitoyl-2-oleoyl-sn-glycero-3-phosphocholine), POPG (1-palmitoyl-2-oleoyl-sn-glycero-3-[phospho-rac-(1-glycerol)]) and DPPG (1,2-dipalmitoyl-sn-glycero-3-[phospho-rac-(1-glycerol)]). The titration of POPC liposomes, as representatives of the eucaryotic membrane model, to lipopeptide solutions showed either no or very weak interactions undetectable by the ITC technique. In turn, the experiments indicated that the lipopeptides interacted with the negatively charged POPG liposomes, with the exception of all the conjugates with lauric acid (Figures S4 and S5, Supplementary Materials). The partitioning of the lipopeptides into the POPG liposomes produced an exothermic effect. A detailed analysis of the binding isotherm confirmed the spontaneous (Δ*G* < 0) and entropy-driven (|TΔ*S*| > |Δ*H*|) process with the binding constant of the order of 10^4^–10^6^ M^−1^ (Table 3).

Shortening of the fatty acid chain length with identical peptide sequence resulted in membrane binding with a less negative enthalpy (Δ*H* = −0.37 ± 0.03 and −0.09 ± 0.01 kcal/mol for C_16_-KKKK-NH_2_ and C_14_-KKKK-NH_2_, respectively). Unexpectedly, the binding constant of C_14_-KKKK-NH_2_ to POPG is two orders of magnitude higher than that determined for C_16_-KKKK-NH_2_. In turn, the titration of C_12_-KKKK-NH_2_ with POPG vesicles resulted in more-or-less constant endothermic effects with no binding saturation. Interestingly, replacing a single lysine with arginine in the C_12_-conjugates induced a weak and constant heat release in the ITC experiment, but still with no binding saturation. The absence of a binding effect in ITC isotherms of both lauric-acid-modified derivatives is compatible with their relatively low antimicrobial potency. The enthalpy of binding to POPG liposomes also became more negative with a single substitution of lysine with arginine residue in the case of C_14_-derivatives, the effect being more pronounced when the arginine was situated closer to the N-terminus. This phenomenon results from the ability of the guanidinium entity of arginine to form a stable bidentate hydrogen bond with the phosphate groups of the membrane lipids [44,45], which strengthens the exothermic contribution to the binding process. Interestingly, stoichiometry of the reaction of ca. 4 for all the myristoyl peptides corresponded to total charge compensation, which suggests that all POPG lipids were accessible to the lipopeptide binding. This result indicated that either the vesicle was destroyed or the peptide was able to translocate into the interior of the vesicle.

The ITC experiments with DPPG lipids were also carried out for the tetralysine series of the lipopeptides (Figure S6, Supplementary Material). Owing to the presence of two saturated palmitoyl hydrocarbon chains, DPPG has a higher main phase transition temperature than POPG does, and at 298 K, it appears in an ordered gel phase. Although biological membranes are fluid in nature, the gel-state membranes are more useful to monitor the event associated with electrostatic binding [46]. All three peptides interacted with DPPG lipids with similar binding constants (Table 3). However, only binding of C_16_-KKKK-NH_2_ to DPPG in the gel phase was accompanied by heat release. The complexation of two remaining lipopeptides, C_14_-KKKK-NH_2_ and C_12_-KKKK-NH_2_, with the gel-state DPPG is endothermic, providing an unfavourable enthalpic contribution (Δ*H* > 0) to the free energy of binding. On the other hand, a large favourable entropy change (TΔ*S* > 0) accompanied this process. This phenomenon is intriguing because shortening the hydrophobic hydrocarbon chain of the fatty acid should intensify the electrostatic effect and, consequently, the exothermic contribution to binding of the peptide to the membrane [47]. However, thermodynamics of the membrane binding depend on two simultaneous processes—the formation of noncovalent bonds and solvent reorganisation. Generally, the former is exothermic, while the latter is endothermic in nature. In the complexation of DPPG with C_14_-KKKK-NH_2_ and C_12_-KKKK-NH_2_, a large positive entropy change clearly indicates a disorder of the molecules upon complex formation, probably arising from the release of a large amount of hydration water from the binding interface or dissociation of water from a more ordered initial state.

### 2.6. Effect of Net Peptide and Counterions on Lipopeptide-Lipid Interactions

The results of our previous study on C_16_-KKKK-NH_2_ showed no effects on the ITC thermogram, which was interpreted as compensation for electrostatic and hydrophobic interactions after binding to the anionic membrane [36]. This phenomenon was further confirmed by FTIR analysis and was also noticed earlier for pentalysin in other studies [48]. In this research, the ITC experiment with C_16_-KKKK-NH_2_ was repeated to compare the results with other lipopeptides tested. This time, the ITC experiment unambiguously confirmed the exothermic reaction to occur at concentrations of the reagents similar to those used previously. Seemingly, the discrepancy with the previous test was different contents of the peptide in the lyophilisates. The freeze-dried peptide powder not only consists of the peptide but also includes non-peptidic components such as water, traces of other solvents, counterions and salts. In turn, the net peptide content is related to the actual weight percent of the peptide in the sample and falls in the range of 60–90 % in most cases, depending on the purity, sequence and method of synthesis and purification [49,50,51,52]. With a very low initial concentration of the peptide used in ITC experiments, the difference in the net peptide content can have a great impact on the stoichiometry. Moreover, the too-low actual concentration of the peptide in relation to the assumed one can lead to an undetectable heat change during the ITC experiment and erroneously indicate the absence of binding. This hypothesis is all the more probable that interactions of C_16_-KKKK-NH_2_ with POPG liposomes are characterised by a low binding constant as compared to those of other lipopeptides (K_ITC_~10^−4^ M^−1^), as shown by the results obtained in this study. Moreover, the results obtained with another compound, C_16_-KGK-NH_2_, also suggest the impact of the peptide lots on the ITC experiments. The ITC experiments carried out previously [36] and now show the same enthalpy of interaction and the binding constant, in spite of the fact that they differ in stoichiometry. In the experiment carried out in this work, the twice-lower stoichiometry is probably due to a higher net peptide content in the lyophilized sample.

Furthermore, we investigated the effect of the content of trifluoroacetate counterions on the interactions between C_16_-KKKK-NH_2_ and POPG liposomes. For this purpose, a simple experiment was carried out in which the concentration of trifluoroacetate ions in the peptide solution was gradually increased. All the peptide solutions were prepared from the same lot of the peptide free of counterions. The buffered solutions of the peptide at a concentration of 0.05 mM containing sodium trifluoroacetate in peptide-sodium trifluoroacetate molar ratios of 1:0, 4:1, 2:1 and 1:1 were titrated with POPG LUVs (Figure S7, Supplementary Material). The results showed that the addition of sodium trifluoroacetate only slightly reduced the enthalpy of peptide–lipid interactions but significantly increased the stoichiometry of the reaction with no effect on the binding constant. It should be emphasised that the control titration of POPG into solution containing only trifluoroacetate ions did not induce any energetic effects.

**Figure 3:**
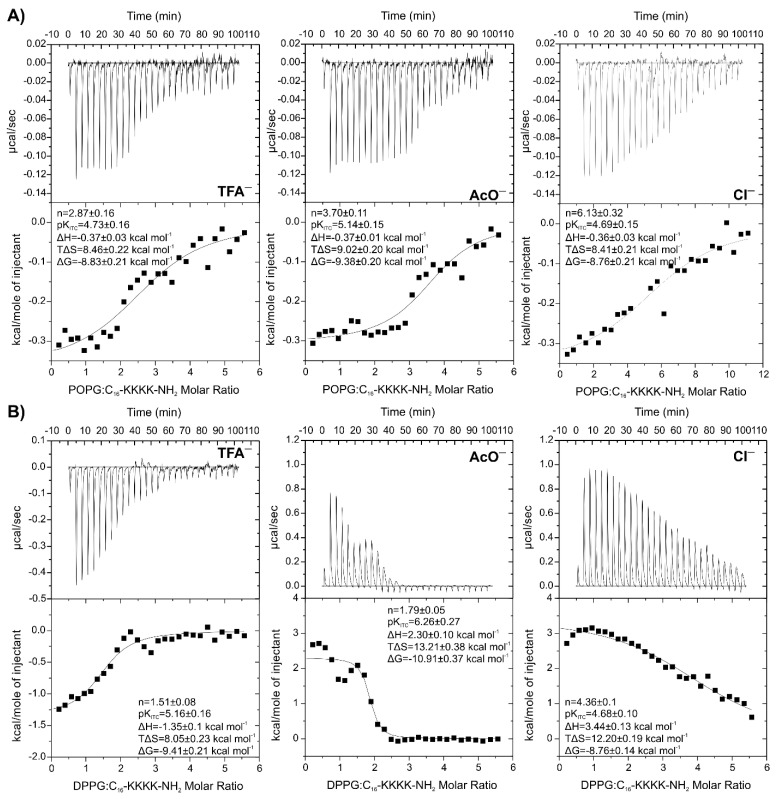
ITC traces showing the heat changes upon titration of (**A**) 1.3 mM POPG to 0.05 mM or 0.025 mM C_16_-KKKK-NH_2_ solutions and (**B**) 1.3 mM DPPG to 0.05 mM C_16_-KKKK-NH_2_ solution at 298.15 K after exchange of the counterions from trifluoroacetate (TFA^−^) to acetate (AcO^−^) and chloride (Cl^−^). The bottom curves represent the heat of reaction vs. the lipid/lipopeptide molar ratio.

The influence of the TFA^−^ content on the thermodynamic parameters of the binding inspired us to check the effect of different counterions (acetate and chloride ions) on the interactions between C_16_-KKKK-NH_2_ and negatively charged liposomes (Figure 3). Regardless of the counterion used, the enthalpies of binding and the binding constants with POPG liposomes were comparable. However, drastic differences were found in stoichiometry of the binding (Table 3), which increased in the order TFA^−^ < AcO^−^ < Cl^−^.

In order to rule out the influence of different net peptide content on the stoichiometry, the relative peptide content in samples with different counterions was estimated. The content was determined spectrophotometrically following the procedure described elsewhere [53]. Mean values of the calculated mass fraction, relative standard deviations (RSD), and percentage contents are presented in Table 4. The percentages were calculated in relation to a sample without counterions, for which the peptide content was assumed to be 100%. The lowest peptide content was determined in the sample with trifluoroacetate ions, whereas the highest one was for that with chloride ions.

If the reaction stoichiometry was only dependent on the peptide content in the lyophilisate, the correction of initial peptide concentration would have led to similar *n* values, irrespective of the counterion nature. However, after taking into account the actual peptide content in the sample and re-fitting one set of site models to the experimental data, the *n* values increased to 4.48 ± 0.27, 5.39 ± 0.17 and 8.20 ± 0.43 in systems titrated with POPG and to 2.61 ± 0.15, 2.56 ± 0.06 and 5.90 ± 0.17 in systems titrated with DPPG, for the samples with TFA^−^, AcO^−^ and Cl^−^ ions, respectively. The results approved the thesis that the nature of counterion also plays a key role in the peptide–lipid interactions. It should be emphasised that the stoichiometry of interactions between peptide and lipid vesicles are averaged over a broader stoichiometry range. However, an *n* value lower than the nominal charge of ~+4 of the C_16_-KKKK-NH_2_ indicates that the lipopeptide is either able to penetrate across the lipid membrane and interact with the inner leaflet or peptide aggregation traps the peptide molecules and hinders its interaction with lipids. In turn, the *n* value twice higher than the nominal charge of the peptide suggests that the peptide binds exclusively to the outer leaflet of POPG LUVs. Deviation from ideality is likely to result from charge screening, steric hindrance, and the formation of a lipid–peptide superstructure. Therefore, the stoichiometry around 8 found for the interaction between the peptide acetate and POPG after correction of the peptide concentration suggests that the peptide remains on the vesicle surface. ITC experiments repeated with DPPG liposomes showed even more pronounced differences between the sample containing TFA^−^ and those with other counterions. As shown in Figure 3B, replacement of trifluoroacetic ions with acetic and chloride ones has a significant effect on the nature of the peptide–lipid interactions. Thus, titration of the gel-state DPPG lipids to the peptide trifluoroacetate resulted in heat release, whereas with two remaining cases, heat absorption occurred. The measured overall enthalpy in the ITC experiment is a sum of negative heat changes accompanying the conformational transition of the peptide upon membrane binding, negative heat changes associated with the formation of new noncovalent bonds such as hydrogen bonds, electrostatic, or van der Waals interactions, positive heat changes associated with desolvation and release of the ordered water molecules from both peptide and membrane surface, and finally, positive heat changes associated with perturbations of the lipid membrane structures as due to peptide binding [54]. Both the endothermic and entropy-favoured process of binding of the C_16_-KKKK-NH_2_ acetate and chloride to the gel-state DPPG show that hydrophobic interactions are crucial for association resulting from an increase in the rotational and translational degrees of freedom of the water molecules following desolvation of the hydrophobic part of the peptide and the release of water and ions from the charged residues. Stronger binding to the lipid bilayer is usually associated with the release of more water molecules, this providing the beneficial contribution of entropy [47]. This phenomenon is distinct in the case of the C_16_-KKKK-NH_2_ acetate with the highest binding constant (K_ITC_~10^6^ M^−1^) and entropic contribution (13.21 ± 0.38 kcal/mol) in free energy as compared to that of the remaining salts. Moreover, as compared to the liquid phase lipid bilayer, the lipid molecules in the gel phase are more tightly packed; hence, relatively more energy or more work must be expended to separate the hydrocarbon lipid chains to enable penetration and binding of the peptide [54]. This can also be considered as a positive contribution to the overall enthalpy of binding of the C_16_-KKKK-NH_2_ acetate and chloride to the gel-state DPPG. By the way of these considerations, the result obtained for the C_16_-KKKK-NH_2_ trifluoroacetate seems to be interesting. The overall exothermic nature of the peptide binding to the gel-state DPPG indicates the predominant role played by electrostatic interaction. Differences between various C_16_-KKKK-NH_2_ salts probably result from ion-specific effects on the peptide in terms of the Hofmeister series. Thus, TFA^−^ belongs to weakly hydrated chaotropes, which are unable to effectively organize water around themselves and seemingly to transfer water molecules to the peptide, thus facilitating its hydration. In contrast, the strongly hydrated kosmotropes, such as AcO^−^ and Cl^−^, are assumed to be able to organize a significant number of water molecules around themselves and effectively extract water from peptide molecules. The weakly hydrated chaotropic anions interact with the peptide bond, whereas the strongly hydrated kosmotropic anions are repelled from it, a phenomenon consistent with the Hofmeister ordering series [55,56]. Therefore, Hofmeister salts are believed to change the hydrophobic/hydrophilic balance of the peptide–water interfaces, i.e., kosmotropes make them more hydrophobic, while chaotropes make them more hydrophilic [57]. On the other hand, anions interacting with positively charged side chains follow the reverse Hofmeister series and strongly hydrated anions interact more strongly than less hydrated ones [56]. Coulombic forces are known to attract counterions to the peptide surface and screen part of the surface charges of the peptides. Consequently, the electric field around the peptide is reduced, thus affecting the initial electrostatic attraction between the peptide and membrane. The weaker binding of the TFA^−^ counterions results in less-effective shielding of the peptide charge and is likely to increase electrostatic attraction between the peptide and membrane. Moreover, the release of the more weakly bound ions from the peptide surface during the membrane binding should be accompanied by a lower entropy gain. To sum up, the results of all those processes taking place during binding of the C_16_-KKKK-NH_2_ to the gel-state DPPG lead to favourable changes in negative enthalpy and/or positive entropy.

### 2.7. Binding of the Peptides to LPS

**Figure 4:**
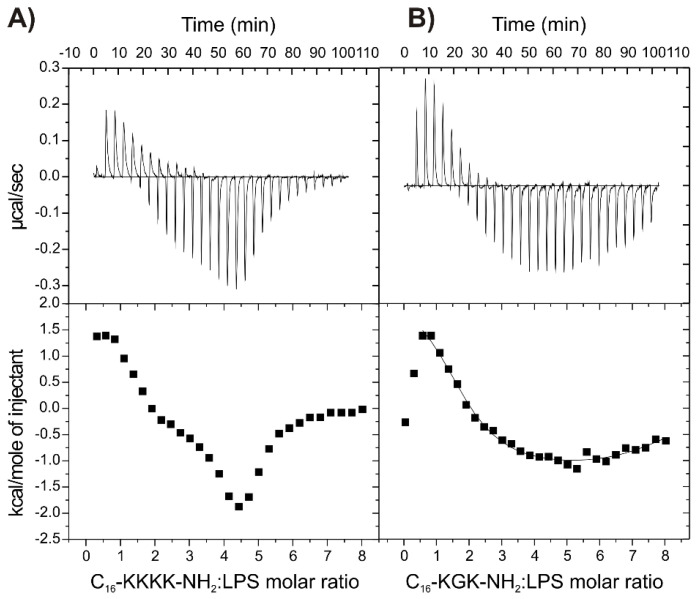
Isothermal titration calorimetry (ITC) traces showing heat changes upon titration of the 0.5 mM C_16_-KKKK-NH2 (**A**) and 0.75 mM C_16_-KGK-NH2 (**B**) to 0.02 mM (400 µg/mL) LPS *E. coli* 055:B5. The bottom curves represent the heat of reaction vs. lipopeptide/LPS molar ratio.

To better understand the increased activity of some lipopeptides against Gram-negative bacteria, their interactions with lipopolysaccharide (LPS) were examined by means of ITC (Figure 4). LPS covers the outer leaflet of the Gram-negative bacterial cell membrane and forms a barrier against external factors. This endotoxin initiates an increased regulation of pro-inflammatory cytokine production and causes septic shock syndromes in humans [58]. Two representative compounds with the best potential against the Gram-negative bacteria, C_16_-KKKK-NH_2_ and C_16_-KGK-NH_2_, were selected for the study of interactions with LPS. As is seen in Figure 4, the heat profiles obtained for both compounds exhibit similar characteristics and indicate complex interactions involving more than a single binding process. In both cases, the titrations began with an endothermic reaction. However, after 4–5 injections, subsequent endothermic effects were accompanied by increasingly stronger exothermic effects, and finally, only an exothermic reaction occurred. The endothermic effects are related to the disruption of the ordered water structure as well as the release of ions from the LPS surface, including calcium ions cross-linking adjacent LPS molecules. This leads to exposition of the negative charges of the LPS and facilitates penetration of the peptide across the bacterial outer membrane [59,60]. In turn, the endothermic–exothermic transition noticed in a single titration is likely to be associated with changes in the lipid phase properties. Based on the previous study [60], it can be speculated that there is a decrease in the melting point of LPS upon association with the lipopeptide.

While ITC experiments showed that both compounds interact with LPS, the calculation of accurate binding affinity (K_ITC_) values for multiple binding events of numerous affinities was impossible for C_16_-KKKK-NH_2_. Again, in the experiment with C_16_-KGK-NH_2_, fitting a two-sets-of-sites model confirmed two separate sequential processes to occur, endothermic and exothermic (Table 3). The negative value of the Gibbs free energy, Δ*G*, and |Δ*H*| < |TΔ*S*| indicated that both processes are spontaneous and entropically driven, but the contribution of entropy to Gibbs free energy is distinctly higher for the former, suggesting that it is more hydrophobic in nature than the latter.

### 2.8. The Effect of Lipopeptide Binding on the Lipid Acyl Chain Order

**Figure 5:**
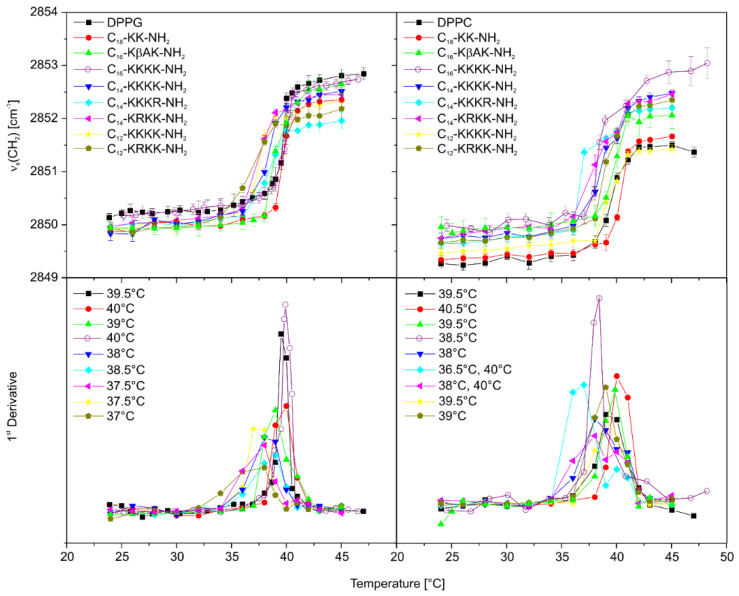
Changes in the ν_s_ (CH_2_) stretching vibrations vs. temperature before and after addition of the lipopeptides in a 10-fold deficiency to DPPG (left) and DPPC (right).

FTIR spectroscopy was employed to obtain detailed information on changes in the membrane structure induced by the lipopeptide binding. For this purpose, frequencies of the CH_2_ symmetric stretching vibrations (ν_s_ ~2850 cm^−1^) of the DPPG and DPPC (1,2-dipalmitoyl-sn-glycero-3-phosphocholine) phospholipids sensitive to order of the hydrocarbon chains were monitored as a function of temperature (Figure 5). The first derivatives of the relationships enabled more precise detection of the main transition temperature (T_m_), required to induce change in the lipid’s physical state from the ordered gel to the liquid-crystalline phase. As has been shown, the lipopeptides reduced T_m_ of the negatively charged DPPG by ca. 0.5–2 °C (except for C_18_-KK-NH_2_), with more evident changes caused by compounds with shorter fatty acid chains. This phenomenon can be easily traced on the example of lipopeptides with tetra-L-lysine in the headgroup, where T_m_ in the DPPG/C_16_-KKKK-NH_2_ complex dropped to a markedly lesser extent than in the DPPG/C_14_-KKKK-NH_2_ and DPPG/C_12_-KKKK-NH_2_ complexes (ΔT_m_ = T_m DPPG-lipopeptide_ − T_m DPPG_ = 0.5, −1.5 and −2 °C, respectively). Shortening of the fatty acid hydrocarbon chains of the lipopeptides elevated the fluidity of the membrane by reducing hydrophobic interactions between the lipids, because the lipopeptides flow laterally and transiently within the lipid bilayer, a phenomenon consistent with the effect of short-chain free fatty acids on the membrane [61,62]. A single replacement of lysine with arginine within the peptide headgroup induced only minor changes as compared to those of the tetra-L-lysine counterparts. Regardless of the changes in T_m_ of DPPG induced by the lipopeptides, all the compounds (except for C_16_-KKKK-NH_2_) more or less affected the order of the lipid acyl chains in both the gel and liquid-crystalline phases. This effect manifested itself by a shift of the CH_2_ stretching bands towards lower wavenumbers as compared to those of neat DPPG. The decrease in the CH_2_ stretching frequency is commonly related to the increased order of the acyl chains. Electrostatic screening of the PG headgroups by oppositely charged compounds reduces electrostatic repulsion between adjacent lipids leads to closer contact between their acyl chains and, consequently, their higher ordering. However, the higher order of the acyl chains should go hand in hand with the elevation of the melting point of the lipids, the effect missing in the case of the lipopeptides. Consequently, a decrease in the stretching frequencies in these cases might be triggered by an enhanced interchain vibrational coupling caused by restriction of the rotational motion of the acyl chains, induced by the lipopeptide headgroup interactions. With a zwitterionic DPPC membrane, the vast majority of the compounds shifted the methylene stretching vibrations towards higher wavenumbers over the entire temperature range as compared to those of the uncomplexed membrane. This phenomenon together with a decrease in T_m_ of the lipids upon lipopeptide binding are related to enhanced fluidity of the membrane. Exceptions to this rule were C_12_-KKKK-NH_2_ and C_18_-KK-NH_2_. Both peptides did not induce significant changes in the frequency of the methylene stretching band over the entire temperature range. Moreover, the former showed no effect on the main DPPC phase transition temperature, while the latter was the only one to increase it by ca. 1 °C.

**Figure 6:**
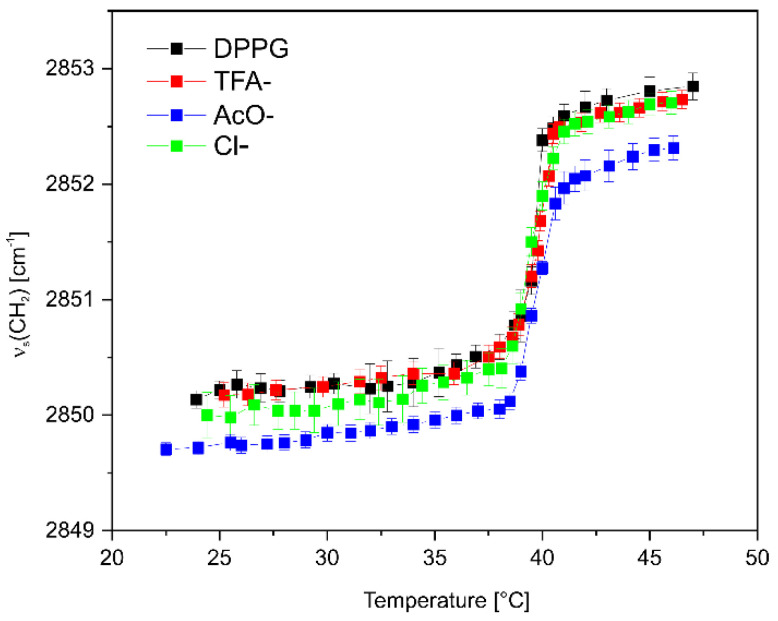
Frequency changes in the ν_s_ (CH_2_) stretching vibrations of DPPG vs. temperature before and after addition of C_16_-KKKK-NH_2_ with various counterions.

Encouraged by the ITC results for various C_16_-KKKK-NH_2_ salts, we performed the FTIR measurements to determine their effect on the DPPG bilayer. As is shown in Figure 6, no changes in the phase transition temperature were seen for any of the samples. However, the presence of AcO^−^ counterion resulted in a distinct decrease in the wavenumber of the CH_2_ symmetric stretching band over the whole temperature range as compared to that of neat DPPG. This phenomenon, along with the missing change in main transition temperature, suggests an enhanced interchain vibration coupling due to restricted rotation of the acyl chains rather than decreased membrane fluidity.

### 2.9. MD Simulations

Highly cationic antimicrobial peptides are expected to target anionic bacterial membranes since electrostatic interactions drive the peptide–membrane association followed by hydrophobic interactions that facilitate anchoring of the peptide to the lipid bilayer. Regardless of a further membrane perturbation model, disruption events occur only upon reaching the threshold concentration of the membrane-bound peptide. Moreover, covering the anionic membrane with the positively charged peptide molecules leads to neutralisation of the membrane surface. This phenomenon is expected to severely affect the membrane function, including its integrity [63,64]. To illustrate the effect of the gradual increase in lipopeptide concentration on the membrane, the coarse-grained molecular dynamics simulations (CG MD) were performed for C_16_-KKKK-NH_2_ interacting with the membranes of Gram-positive (3:1 POPG/POPE) and Gram-negative bacteria (RaLPS—Ra mutant rough chemotype lipopolysaccharide—in the outer leaflet and 18:1:1 POPE/POPG/CDL2 in the inner leaflet, CDL2—cardiolipin 2). The CG MD simulations were divided into several consecutive parts, in which an excess of lipopeptide molecules was added to the system at the water–membrane interface. At the same time, lipopeptide molecules that freely moved away from the outer leaflet of the membrane on account of initial crowding and were able to bind to the inner leaflet due to periodic boundary conditions were removed from the system.

**Figure 7:**
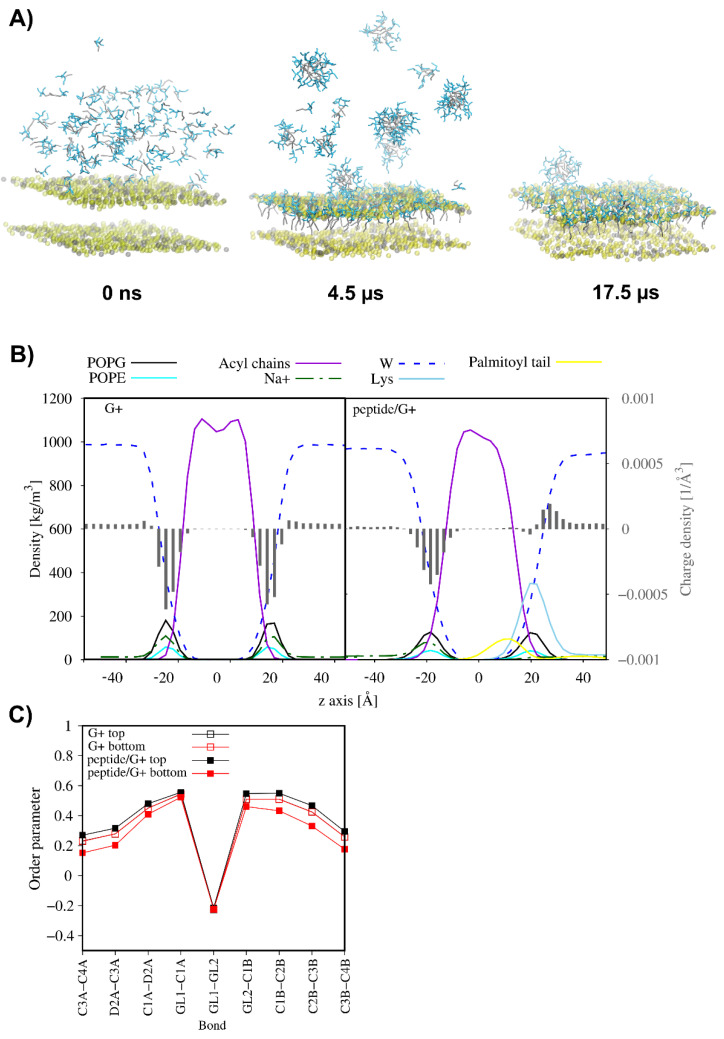
Representative snapshots from the 3:1 POPG/POPE binding CG MD simulations for C_16_-KKKK-NH_2_. For clarity, only phosphate beads of the membrane have been displayed. The POPE and POPG phosphate groups are indicated in gray and yellow, respectively. Palmitoyl tails are in gray, while lysine residues are in cyan (**A**). Partial density and charge density profiles averaged over the last 100 ns of the CG MD simulations (**B**) and comparison of the lipid acyl chain order parameters of the lipids in the outer and inner leaflet of the membrane in the absence of the lipopeptide and in the presence of the lipopeptide (**C**).

**Figure 8:**
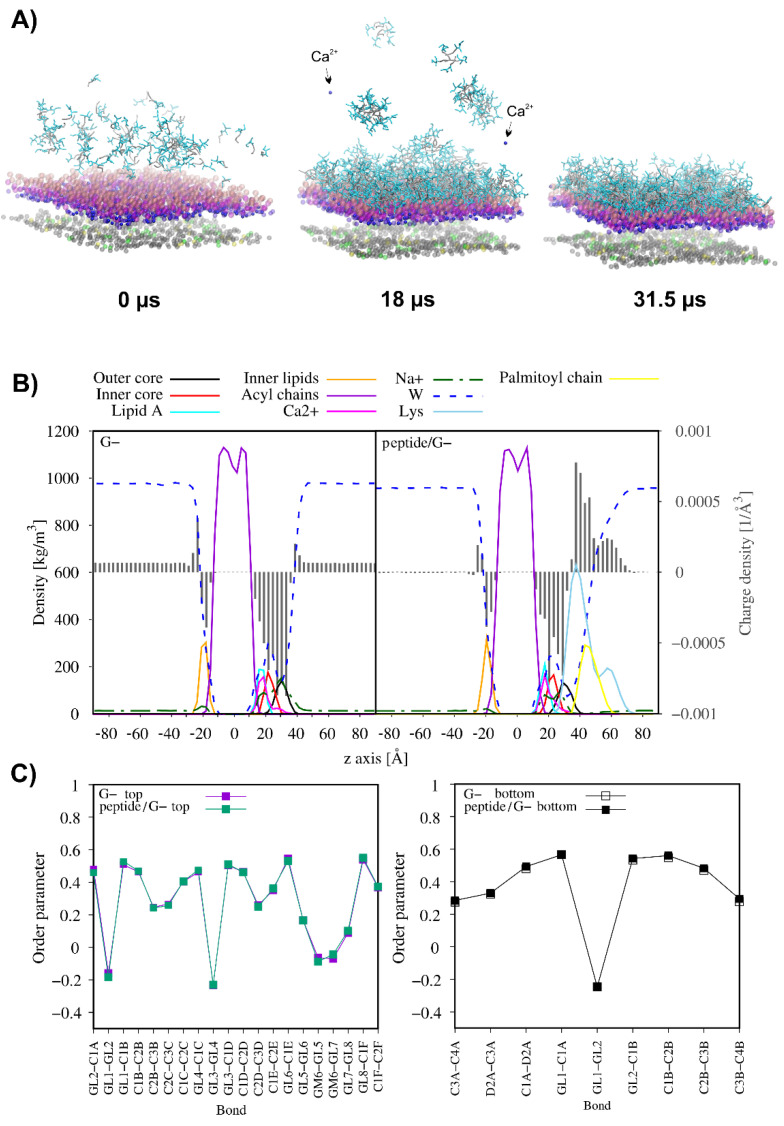
Representative snapshots from the Ra mutant rough chemotype lipopolysaccharide RaLPS/POPE/POPG/CDL2 binding CG MD simulations for C_16_-KKKK-NH_2_. For clarity, only negatively charged beads of the membrane have been displayed. Phosphate groups of the outer oligosaccharide core and Lipid A of RaLPS are in pink and in violet, respectively. The acid groups within the inner oligosaccharide domain of RaLPS are in magenta. Phosphate groups of the inner POPE, POPG, and CDL2 are in gray, yellow and green, respectively. Palmitoyl tails are in gray, while lysine residues are in cyan. The arrows indicate Ca^2+^ ions released from the Lipid A part of RaLPS during simulations (**A**). Partial density and charge density profiles averaged over the last 100 ns of CG MD simulations (**B**) and comparison of the lipid acyl chain order parameters of the RaLPS and POPE and POPG in the systems in the absence of the lipopeptide and presence of the lipopeptide (**C**).

The results of CG MD simulations suggest that C_16_-KKKK-NH_2_ adsorbed rapidly on the outer surface of both membranes. However, as is shown in Figure 7 and Figure 8, the lipopeptide readily entered into the 3:1 POPG:POPE membrane core but showed only a limited tendency towards insertion into the RaLPS layer of the asymmetric membrane. With each successive step, as the outer leaflet of the membrane was gradually saturated with the positively charged lipopeptide molecules, the tendency to attract more peptide molecules to the membrane surface drastically decreased. Finally, a total of 121 and 292 C_16_-KKKK-NH_2_ molecules were attached to the outer leaflet of the POPG:POPE and the RaLPS:POPE:POPG:CDL2 membranes, respectively, corresponding to the charge ratios of 1.3:1 and 1:1.6 (defined as the number of positive charges from the lipopeptide per negative charge from the lipids in the outer leaflet). As a consequence, the electrical state of the outer membrane surface in both models changed radically as compared to those of peptide-free membranes ( Figure 7B and Figure 8B). In the former, overcharging of the originally negatively charged POPG:POPE membrane by the positively charged lipopeptide was even noticed. This phenomenon forced the displacement of sodium ions from the membrane–water interface and led to the loss of electric charge balance on both sides of the membrane. Consequently, the electrostatic potential across the membrane became disturbed to affect bacterial cellular behaviours and functions. It is worth noting that in asymmetric RaLPS-coated membrane, adsorption of the C_16_-KKKK-NH_2_ also led to the release of single calcium ions from the Lipid A region (Figure 8). Salt bridges formed by calcium ions and adjacent LPS molecules are critical to the structural integrity of the membrane, revealing the disruptive nature of the electrostatic repulsive forces between LPS molecules when the charge screen is removed [65].

As just mentioned, C_16_-KKKK-NH_2_ was fully transferred into the POPG:POPE membrane. This phenomenon drastically reduced the average area per lipid (APL) in the outer leaflet and only slightly increased it in the inner leaflet (Table 5). Interestingly, binding of the lipopeptide had no overall effect on the lateral diffusion of the lipids in the peptide-bound leaflet but increased the lateral diffusion of the POPG in the internal leaflet ca. 1.4-fold as compared to that of the peptide-free system (from 4.56 ± 0.64 × 10^−7^ to 6.46 ± 0.79 × 10^−7^ cm^2^/s). This change is compatible with a decrease in the order parameters of the acyl chains of the inner lipids (Figure 7C) and reflects the greater fluidity of the inner leaflet of the membrane. In turn, in the membrane model of Gram-negative bacteria, where the lipopeptide remained adsorbed on the membrane surface, only a slight decrease in APL of RaLPS and no unambiguous changes in APL values for the lipids in the bottom leaflet were noticed. Moreover, binding of the lipopeptide had no effect on the lateral diffusion of the RaLPS but accelerated lateral diffusion of the POPG and reduced lateral diffusion of POPE and CDL2 in the internal leaflet of the RaLPS:POPE:POPG:CDL2 membrane. Generally, however, these changes did not correlate with membrane fluidity because the order parameters remained unchanged upon lipopeptide adsorption.

## 3. Materials and Methods

### 3.1. Reagents

1,2-dipalmitoyl-sn-glycero-3-phosphoglycerol (DPPG), 1,2-dipalmitoyl-sn-glycero-3-phosphocholine (DPPC), 1-palmitoyl-2-oleoyl-sn-glycero-3-phosphoglycerol (POPG) and 1-palmitoyl-2-oleoyl-sn-glycero-3-phosphocholine (POPC) were acquired from Avanti Polar Lipids, Inc. (Alabaster, AL, USA). *Escherichia coli* ATCC 25922, S*taphylococcus aureus* ATCC 25923, *Pseudomonas aeruginosa* ATCC 9027, *Staphylococcus epidermidis* ATCC 14990, and *Candida albicans* ATCC 10231 were obtained from Polish Collection of Microorganisms, Wroclaw, Poland. The phosphate-buffered solution (10 mM phosphate buffer, pH 7.4, containing 2.7 mM potassium chloride and 137 mM sodium chloride) was purchased from Sigma Aldrich (Darmstadt, Germany).

### 3.2. Lipopeptide Synthesis

Lipopeptides were synthesized manually on polystyrene resin with RAM linker (Fmoc-Rink-Amide-AM resin, loading ca. 1.0 mmol/g; Orpegen Peptide Chemicals GmbH, Heidelberg, Germany) by solid-phase Fmoc/tBu methodology. Amino acids were purchased from Orpegen Peptide Chemicals GmbH (Heidelberg, Germany)—Fmoc-β-Ala-OH, Fmoc-Gly-OH, Fmoc-L-Arg(Pbf)-OH, and Fmoc-L-Lys(Boc)-OH. Fatty acids—dodecanoic acid (C_12_), tetradecanoic acid (C_14_), hexadecanoic acid (C_16_), and octadecanoic acid (C_18_) were acquired from Merck (Darmstadt, Germany). To avoid racemization during acylation, Oxyma Pure (Iris Biotech GmbH, Marktredwitz, Germany) was used. Acylation was conducted with a DIC (Iris Biotech GmbH, Marktredwitz, Germany): OxymaPure: Fmoc-AA-OH mixture (mole ratio 1:1:1) dissolved in N,N’-dimethylformamide (DMF; POCh, Avantor, Gliwice, Poland) and dichloromethane (DCM; Chempur, Piekary Slaskie, Poland) (1:1, *v/v*) in a fourfold excess based on the resin for 90 min. Deprotection of the Fmoc group was performed for 15 min using a 20% piperidine (Iris Biotech GmbH, Marktredwitz, Germany) solution (*v/v*) in DMF. After each acylation and deprotection, the resin was rinsed with DMF and DCM. The presence of free amine groups was confirmed by a chloranil test. For the final deprotection and cleavage of the lipopeptide, the mixture of trifluoracetic acid (TFA; Apollo Scientific, Denton, UK), triisopropylsilane (TIS; Iris Biotech GmbH, Marktredwitz, Germany) and deionized water (95:2.5:2.5, *v/v/v*) was used. Crude lipopeptides were precipitated using cold diethyl ether (POCh, Avantor, Gliwice, Poland). After centrifugation, the sediments were dissolved in acetic acid (Chempur, Piekary Slaskie, Poland) and lyophilised. The lipopeptides were purified by reversed-phase high-performance liquid chromatography (RP-HPLC). Purifications were carried out on a Phenomenex Gemini-NX C_18_ column (21.20 mm × 100 mm, 5.0 µm particle size, 110 Å pore size). UV detection at 214 nm was used, and the crude peptides were eluted with a linear 20–75% acetonitrile gradient (ACN for HPLC-gradient grade; POCh, Avantor, Gliwice, Poland) in deionized water over 60 min at room temperature. The mobile phase flow rate was 10.0 mL/min. Both eluents contained 0.1% (*v/v*) of TFA. Fractions were analysed on a Waters X-Bridge Shield RP-18 column (3.0 mm × 100 mm, 3.5 µm particle size, 130 Å pore size) with UV detection at 214 nm (Varian ProStar HPLC system, Varian, Inc., Palo Alto, CA, USA). Pure fractions (>95%, HPLC) were collected and lyophilised. The identity of synthesised lipopeptides was confirmed by mass spectrometry (ESI-MS; Waters Alliance e2695 system with an Acquity QDa detector; Waters, Milford, MA, USA). The calculated and measured m/z values are listed in the Supplementary Materials (Table S1).

### 3.3. Counterion Exchange

The exchange of TFA^−^ for Cl^−^ was performed as described elsewhere [66]. Briefly, the lipopeptide was dissolved in 0.5% HCl in acetonitrile and incubated for 5 min at RT (lipopeptide concentration was 1 mg/mL). Then, the solvent was evaporated under reduced pressure using a rotary evaporator at 40 °C. The exchange procedure was repeated twice. Finally, the lipopeptide was lyophilised from water to remove the excess chlorides. To obtain the lipopeptide without counterions, the TFA^−^ ions were removed from the sample by using bicarbonate ion-exchange resin (VariPure IPE columns, polymer supported quaternary amine resin with a bicarbonate counter ion; Agilent Technologies Inc., Santa Clara, CA, USA). The exchange of TFA^−^ to AcO^−^ was accomplished in two steps. First, TFA anions were removed in the VariPure IPE column. Subsequently, the dilute acetic acid (1 M) was added, incubated for 5 min, and then lyophilized. Ion chromatography (Dionex ICS-5000+, Thermo-Scientific, Sunnyvale, CA, USA) was used to confirm successful anion exchange. The method was validated for the analysis of TFA^−^, AcO^−^, and Cl^−^ according to the ICH guidelines Q2 (R1) [67]. The analyses were performed with isocratic elution (4.5 mM Na_2_CO_3_ and 1.4 mM NaHCO_3_ in water), a flow rate of 1.2 mL/min, and an injection volume of 20 µL. All the samples were dissolved in water to a concentration of 0.5 mg/mL. Ions were detected by suppressed conductivity with ASRS 300—anion self-regenerating suppressor and the suppressor current of 31 mA. Column characteristics: Dionex IonPac AS22, dimensions 4.0 mm × 250 mm. Column compartment temperature was set at 30 ± 0.1 °C and conductivity detector temperature was 35 ± 0.1 °C. In effect lipopeptide (A) without counterion, as well as with (B) TFA^−^, (C) AcO^−^, (D) Cl^−^ were obtained. For C_16_-KKKK-NH_2_ trifluoroacetate and chloride, only TFA^−^ and Cl^−^ anions were detected, and their levels were 240.63 and 44.8 ppm per mg of the lyophilised sample, respectively. For C_16_-KKKK-NH_2_, the acetate exchange rate and level of acetate ions reached 86.6%, presented as % of µmol of all anions in the sample. Other ions found in this sample were Cl^−^ (8.5%) and TFA^−^ (4.9%).

### 3.4. Microbiological Studies

The minimum inhibitory concentration (MIC) was determined according to the procedure recommended by the Clinical Laboratory Standards Institute guidelines [68,69]. Reference strains of bacteria: *Staphylococcus aureus* ATCC 25923, *Staphylococcus epidermidis* ATCC 14990, *Escherichia coli* ATCC 25922, *Pseudomonas aeruginosa* ATCC 9027, and fungus *Candida albicans* ATCC 10231 were obtained from Polish Collection of Microorganisms (PCM, Polish Academy of Sciences, Wroclaw, Poland). Bacteria at initial inoculums of 5 × 10^5^ CFU/mL in Mueller Hinton Broth (MHB), and fungi at initial inoculums of 2 × 10^3^ CFU/mL in RPMI-1640 were poured onto a 96-well plate at an equal volume and exposed to lipopeptides at increasing concentrations (1–512 µg/mL). The plates with the compounds were incubated for 18 h at 37 °C for bacteria and 48 h at 25 °C for fungi. The minimum inhibitory concentration was assumed as the lowest concentration of a lipopeptide at which the observable growth was inhibited.

### 3.5. Evaluation of Haemolytic and Cytotoxic Activities

The haemolytic activity of the compounds was measured after exposure of human red blood cells to the lipopeptides at graded concentrations. The lipopeptides were dissolved in a phosphate buffer. Red blood cells taken from a healthy donor were separated from plasma by centrifugation. Then, they were washed three times in PBS, centrifuged, and resuspended in PBS. The erythrocytes were incubated with different concentrations of lipopeptides at 37 °C for 1 h and centrifuged (4 °C, 5 min, 1000× *g*). The supernatants were transferred to 96-well plates and haemoglobin release was measured with a microplate reader (Multiskan™ GO Microplate Spectrophotometer, Thermo Scientific, Waltham, MA, USA) by recording the absorbance at 550 nm. A 0.1 % Triton X-100 solution was used as a positive control, and pure PBS was used as a negative one.

The cytotoxicity of the compounds was evaluated on the basis of a tetrazolium salt reduction test (MTT) performed along the lines of human keratinocytes HaCaT. The keratinocytes were planted in 96-well plates in a medium supplemented with serum. After 24 h, the medium was changed to a serum-free DMEM one containing graded concentrations of peptides. The cells were incubated with peptides for 48 h. Thereafter, MTT was added in order to reach a final concentration of 0.5 mg/mL, and the plates were incubated at 37 °C for 4 h. Then, the MTT reading was analysed using a plate reader. The absorbance at 630 nm (background absorbance) was subtracted from that at 570 nm for each well.

### 3.6. Serum Stability Studies

The serum stability of peptides was tested in human serum (human serum, from human male AB plasma, Sigma-Aldrich, Darmstadt, Germany). At first, the 25% serum was centrifuged at 14,800 rpm for 10 min to remove the excessive amount of lipids in the serum preparation, and the supernatant was collected and incubated at 37 °C for 15 min under shaking. The assay followed the addition of the peptide to the serum up to a final peptide concentration of 75 μg/mL. The 50 μL aliquots of the incubated mixtures were taken at the following time intervals: 0, 30, 60, 120 and 240 min. The aliquots were mixed with 50 μL of trichloroacetic acid (final concentration 3% of TCA), incubated on ice for 15 min to precipitate serum proteins, and then centrifuged (14,800 rpm, 10 min). The supernatants were transferred to glass vials and stored at 4 °C until analysis. As a control, a 25% serum treated as just mentioned was used and collected under the same time intervals. The assays were performed in triplicate. RP-UPLC analysis was performed using a Nexera system (Shimadzu Europe GmbH, Duisburg, Germany) with a C12 Jupiter Proteo column (150 mm × 2 mm, 90 Å, 4 micron) (Phenomenex Inc., Torrance, CA, USA) applying a linear aqueous acetonitrile gradient 1–80% B in 20 min and 1–90% B in 20 min for peptides with C_14_ and C_16_ fatty acid chains, respectively, using solutions A (0.1% TFA in water) and B (0.1% TFA in acetonitrile) with a flow rate 0.6 mL/min and detection at 220 nm.

### 3.7. Critical Aggregation Concentration Measurements

Critical aggregation concentrations (CACs) were determined in the phosphate-buffered solution at 298 K using a K100 Force tensiometer equipped with two micro-dispensers (Krüss GmbH, Hamburg, Germany) by measuring the surface tension (SFT) of each lipopeptide concentration series. After each dilution, the samples were stirred with a magnetic stirrer. The surface tension measurements were carried out by the Wilhelmy plate method. The platinum plate was thoroughly cleaned and flame dried before use. The SFT measurements were repeated ten times for each concentration but only five items with the lowest standard deviation were selected and averaged. The standard deviation did not exceed 0.1 mN/m. The critical aggregation concentration was defined as the intersection of two lines fitted to the measuring points before and after reaching the CAC value.

### 3.8. Circular Dichroism Spectroscopy

The CD spectra were recorded at 298 K using a Jasco J-815 spectropolarimeter. The measurements were conducted on 0.15 mg/mL peptide solutions over the range of 185–260 nm with a 1 mm path length. The spectra were corrected by subtracting the background from the sample spectrum and plotted as a mean molar ellipticity per residue (MRME, degree × cm^2^ × dmol^−1^) vs. wavelength λ (nm). The signal/noise ratio was increased by acquiring each spectrum over an average of three scans.

### 3.9. Thioflavin T (ThT) Assay

The peptide was dissolved in Eppendorf Low Retention Tubes in 1.0 mL of PBS, pH 7.4, at a concentration of 1.0 mg/mL and incubated at 37 °C with constant orbital shaking. Aliquots for the evaluation of the incubation were taken before incubation and after 1–25 h. In the period up to 2 h, the samples were measured every 15 min and afterwards every 30 min. 20 µL of the peptide solution was mixed with 10 μL of 1.6 mM Thioflavin T (ThT) in water and 90 µL of PBS pH 7.4. The solution of the 1.6 mM ThT was used as the dilution standard (blank) in the experiment. The fluorescence was measured on a Tecan Infinite 200Pro spectrofluorometer (TECAN, Group Ltd., Männedorf, Switzerland) in 96-well black plates (Corning TM Costar, Camelback RD, AZ, USA), with the excitation wavelength set at 420 nm and emission in the range of 455 to 600 nm.

### 3.10. Transmission Electron Microscopy (TEM)

Peptide samples, at concentrations of 1 and 2 mg/mL, were incubated at PBS pH 7.4, at 37 °C with agitation for 24 h. Peptide solution (5 µL) was applied on a glow-discharged carbon-coated copper grid (400 mesh). After 1 min of adsorption, excess liquid was removed using filter paper, and the samples were stained with a 2% (*v/v*) aqueous uranyl acetate. The samples were examined with a TECNAI SPIRIT BIO TWIN FEI at 120 kV, with nominal magnifications between 11,500 and 39,000. The assay was performed on samples just after dissolution, as well as after 4 and 24 h of incubation.

### 3.11. Fluorescence Spectroscopy

The bacterial viability was determined using imaging by fluorescence microscope (Leica DMI4000B). The selected Gram-positive and Gram-negative bacteria (*Staphylococcus epidermidis* ATCC 14990 and *Escherichia coli* ATCC 25922, respectively) were inoculated in the Luria–Bertani medium and cultured with shaking at 37 °C up to the early exponential phase (A_600_ = 0.1). Afterwards, 1.5 mL of the cultures were transferred to 5 mL Eppendorf tubes and treated with a lipopeptide at concentrations of 1×, 2× and 4× MIC, and with a pure vehicle (water) or 70% ethanol for the negative and positive controls, respectively. Then, bacteria were stained with a LIVE/DEAD™ BacLight™ Bacterial Viability Kit (ThermoFisher Scientific, Waltham, MA, USA) containing two dyes: a bacterial membrane-permeable Syto9 (green fluorescence) and a bacterial membrane-impermeable propidium iodide (red fluorescence), which stains only bacteria with damaged membranes. The details of the experiments were described previously [70].

### 3.12. FTIR Measurements

Fourier-transform infrared spectroscopy (FTIR) was employed to study the interactions of the lipopeptides with liposomes composed either of negatively charged DPPG or zwitterionic DPPC phospholipids. The liposomes were prepared by the thin-film hydration method. For this purpose, DPPG or DPPC phospholipids were dissolved either in a chloroform:methanol (4:1, *v/v*) mixture or chloroform alone, respectively. The solvent was removed under nitrogen and then freeze-dried overnight to remove all traces of it. Afterwards, the dry lipid film was hydrated with a phosphate buffer (pH 7.4) up to a final lipid concentration of 50 mg/mL and incubated in a thermomixer at 60 °C for 2 h. With peptide-lipid samples, the lipopeptide was added to a suspension of phospholipid at a 1:10 molar ratio and incubated for another 2 h at 60 °C. To reduce the lamellarity and the size of the multilamellar vesicles (MLVs), the content of the vial was frozen in liquid nitrogen and alternately heated several times. The resulting suspension was placed between two CaF_2_ windows with a 50 μm Teflon spacer. Measurements were performed on an FTIR spectrometer (IFS66, Bruker, Billerica, MA, USA) equipped with a DTGS detector. The spectra were recorded over the 2500–3000 cm^−1^ range with a resolution of 2 cm^−1^. Each sample was examined over the range of 25–45 °C. The final spectra were the average of 10 measurements, where a single measurement was scanned 16 times. The temperature during the measurement was controlled with a CHY502 thermometer. The phosphate buffer in the reference cell was scanned as the background and subtracted from the spectra of the samples. The data were processed in Origin 2018 (Northampton, MA, USA), and the wavenumber location for symmetric CH_2_ stretching mode was indicated using a multiple Gaussian curve fitting procedure. The data were presented as the wave number of the CH_2_ stretching vibration vs. temperature.

### 3.13. ITC Measurements

The unilamellar lipid vesicles (LUVs) for ITC measurements were formulated from POPG or POPC phospholipids by the previously described procedure [36]. The stock solution of the LPS was prepared by dissolving LPS *E. coli* 055:B5 (Sigma Aldrich, Darmstadt, Germany) in PBS at a concentration of 1 mg/mL. Titrations were performed at 298.15 K using an AutoITC isothermal titration calorimeter (MicroCal Inc., Northampton, MA, USA). All ITC experiments consisted of 29 injections each of 10.02 μL (2 μL for the first injection) of the lipid suspension (1.3 mM) into the calorimetric cell containing 1.4491 mL of buffered lipopeptide solution (PBS, pH 7.4) at a concentration of 0.05 or 0.1 mM with a 4 min interval between injections. Each injection lasted 20 s. In the experiments with LPS, the buffered lipopeptide solution at a concentration of 0.75 mM was gradually added to the reaction cell with LPS (400 µg/mL). The molecular mass of LPS was assumed to be 20,000 Da. To ensure homogeneous mixing in the cell, the stirrer speed was kept constant at 300 rpm. To assess the heat of dilution, control experiments were completed by titrating lipid vesicles (1.3 mM) into a PBS solution. Titration data were processed using the ITC module for ORIGIN 7 software, provided by MicroCal Inc. The binding constants, stoichiometry, and enthalpy of interactions were derived using one of the fitting models provided with the ITC software. The changes in entropy (∆*S*) and the Gibbs free energy (∆*G*) were calculated from the following equation:

∆*G* = −RTln(55.5 *K*_ITC_) = ∆*H* − T∆*S* (1)

where 55.5 is the molar concentration of water, R is the gas constant (1.986 cal × mol^−1^ × K^−1^) and T is the absolute temperature.

### 3.14. Coarse-Grained Molecular Dynamic Simulations

Coarse-grained molecular dynamics simulations were carried out using the MARTINI force field [71,72] implemented in the GROMACS 2019.5 package [73]. The lipid bilayers were built with the CHARMM-GUI web-based graphical interface [74,75,76,77]. Two models of the bacterial membrane were built to mimic the membranes of Gram-positive and Gram-negative bacteria. The former consisted of POPG and POPE lipids at a ratio of 3:1, equally distributed between two leaflets of the membrane [78], with the latter consisting of lipopolysaccharide (RaLPS) molecules in the outer leaflet and a mixture of POPE, POPG and CDL2 (18:1:1) in the inner leaflet [79]. The membranes with counterions (sodium ions to neutralize POPG, CDL2, and the outer and inner core of RaLPS, and calcium ions to neutralize Lipid A) but without water were used to construct the systems with lipopeptide molecules. First, one hundred lipopeptide molecules were randomly distributed above the outer membrane surface using the insert-molecules tool of the GROMACS package. Afterwards, the systems were solvated and the lipopeptide molecules were neutralised with chloride ions. The salt concentration in bulk solution was kept at 100 mM NaCl. The systems were energy-minimised and equilibrated with a stepwise lowered force constant of the harmonic restraints (from 200 to 10 kJ mol^−1^ nm^−2^) to fix the position of the headgroups of the membrane lipids during simulations. Then, both systems were subjected to the isothermal–isobaric molecular dynamics (NTP) with a 10 fs time step, as suggested by Wigner et al. [80]. The temperature was held at 310 K using v-rescale temperature coupling. The pressure was treated semi-isotropically at 1 bar using the Parinello–Rahman barostat with a coupling constant τ_p_ = 12.0 ps. The relative dielectric constant for explicit screening was 15. Coulomb interactions were treated using a reaction-field and a cutoff of 11 Å. In the initial steps of the simulations, some peptide molecules were able to move free away from the outer surface and bound to the inner membrane leaflet under periodic boundary conditions. These molecules along with the close chloride counterions were removed from the systems to reflect the natural conditions, under which the lipopeptide can only interact with the outer membrane surface at the beginning of the interactions. At the same time, further 20–50 lipopeptide molecules were added to the system above the outer layer of the membrane along with the chloride counterions. The systems were once again energy-minimised and equilibrated with position-restrained peptide molecules, using a force constant of 1000 kJ mol^−1^ nm^−2^. Afterwards, the systems were subjected to the 200–2500 ns NTP molecular dynamics simulations without any restraints. Additionally, again, the lipopeptide molecules able to bind to the inner membrane leaflet under periodic boundary conditions were removed and a further 20–50 lipopeptide molecules were added to the systems. The procedure was repeated several times to obtain systems saturated with the lipopeptide. The entire MD simulations were run for 17.5 and 31.5 µs for the Gram-positive and Gram-negative membrane models, respectively.

The data were analysed with standard tools of the GROMACS and GridMAT [81]. The membrane thickness was defined as the distance between the phosphate particles of the lipids in the opposite membrane leaflets. In the case of the RaLPS, the phosphate particles of Lipid A were considered in membrane thickness calculations. The order parameters for Martini lipids were calculated with a do-ordered-gmx5.py script available at www.cgmartini.nl (accessed 27 June 2022). Lipid lateral diffusion coefficients were obtained from the linear-fitted slope of averaged two-dimensional mean square displacement (MSD) from gmx msd, with the initial reference point reset every 1 ns.

## 4. Conclusions

The continual growth of drug resistance among pathogenic microorganisms forces the search for new methods of combating infections. However, research on innovative drugs is costly, time-consuming, and often unsuccessful. In order to increase the chances of success, inspiration comes directly from nature. For instance, antimicrobial peptides, which are important components of the immune system of all living organisms, or their synthetic analogues, which are cheaper and easier to synthesise, are a promising source of new antibiotics. In the framework of this scenario, we continue to research short synthetic antimicrobial lipopeptides, a promising weapon in the fight against microbial infections. The lipopeptides consist of 2–4 amino acid residues (mainly Lys and Arg) coupled with fatty acids of different hydrocarbon chain lengths (C_12_–C_18_). They inhibit the growth of almost all the Gram-positive bacteria, whereas those modified with palmitic acid also reveal promising activity against reference Gram-negative bacteria as well as fungus *C. albicans*. Shortening in the fatty acid hydrocarbon chain drastically reduces the activity against *C. albicans*, while a single substitution of lysine residue with the arginine one intensifies the activity against *S. epidermidis*. All tested lipopeptides show a tendency towards self-assembly over the concentration range of 0.06–3.6 mM in the PBS solution. In most cases, the critical aggregation concentration (CAC) far surpasses the MIC values; apparently, the monomers provide an active form of the lipopeptides. However, it cannot be ruled out that the presence of bacterial cells and interactions with the lipid bilayer affect the concentration at which self-assembly of lipopeptides begins. In this context, the case of C_16_-KβAK-NH_2_ seems to be of particular interest. The CD spectra, along with ThT assay and TEM micrographs, demonstrated unambiguously that C_16_-KβAK-NH_2_ forms a β-like structure. Although the peptide concentrations used in these experiments exceeded the MIC values, characteristic flocs could be noticed, approving the formation of fibrils, also in more dilute solutions. This precluded the determination of the CAC value by means of surface tension measurements. It seems likely that this finding is responsible for a slight drop in the activity against some bacterial strains, but at the same time, it increases the stability of the peptide in the serum and reduces the cytotoxicity against human keratinocytes.

The results of our study clearly indicate that the target of action of lipopeptides is the bacterial membrane. Interestingly, the ITC experiments and the molecular dynamics simulations suggest that the interaction of the ultrashort cationic lipopeptide with the membrane of Gram-negative bacteria leads to the release of calcium ions necessary for the stabilization of the lipopolysaccharide layer.

The previous results showed that removal of the TFA^−^ counterions from the lipopeptide either do not affect the activity or the results differ only slightly [82]. In this study, we evaluate the effect of counterion types on the interaction of the most active compound, C_16_-KKKK-NH_2_, with the bacterial membrane. In all cases, the ITC results confirmed interactions with negatively charged lipids (POPG and DPPG), main components of bacterial membranes. With POPG, the binding process was always exothermic, entropy-driven, and with comparable binding constants, but completely different in stoichiometry, increasing in the order of TFA^−^ < AcO^−^ < Cl^−^. This increase up to a value much surpassing the nominal charge of the peptide indicates that the peptide does not penetrate the interior of the liposome and cannot interact with its inner leaflet, but rather remains bound to the liposome surface. The addition of counterions that interact more strongly with the peptide than do trifluoroacetate anions can alter the degree of penetration of the lipid bilayer. However, this change does not appear to be of major importance for antimicrobial activity. Even more interesting results were obtained in experiments with gel-state DPPG. As was shown, the interaction of the C_16_-KKKK-NH_2_ trifluoracetate with the gel-state DPPG liposomes was exothermic, unlike the other two salts, acetate and chloride, for which an endothermic effect was noticed. Perhaps, the difference is due to specific counterion–peptide interactions and provides a strong incentive to further investigate the role of the counterion in biological activity and membrane interactions.

## Figures and Tables

**Table 1:** Antimicrobial activity of lipopeptides against selected microorganisms and IC_50_ values determined by the tests on human keratinocytes (HaCaT).

Peptide	MIC μg/mL					IC_50_ (μg/mL)
	*S. aureus*	*S. epidermidis*	*E. coli*	*P. aeruginosa*	*C. albicans*	
C_18_-KK-NH_2_	64	4	128	64	4	0.69 ± 0.001
C_16_-KK-NH_2_	8	4	8	128	4	2.5 ± 0.8
C_16_-KKKK-NH_2_	8	8	16	16	16	23.52 ± 1.30
C_16_-KGK-NH_2_	16	4	8	64	8	7.41 ± 3.05
C_16_-KβAK-NH_2_	128	2	64	64	4	25.11 ± 6.17
C_14_-KKKK-NH_2_	32	2	128	64	128	15.63 ± 0.74
C_14_-KKKR-NH_2_	32	≤1	128	128	128	32.5 ± 10.0
C_14_-KRKK-NH_2_	16	≤1	64	64	128	20.38 ± 4.12
C_12_-KKKK-NH_2_	64	16	512	256	>512	-
C_12_-KRKK-NH_2_	64	≤1	512	512	512	-

**Table 2:** Critical micelle concentration and surface tension at CAC measured in a phosphate-buffered solution (pH 7.4).

Peptide	CAC mM	CAC μg/mL	γCAC mN/m
C_18_-KK-NH_2_	0.06	34	45
C_16_-KK-NH_2_	0.09 (1.07)	45	43
C_16_-KKKK-NH_2_	0.75 (14.6)	575	46
C_16_-KGK-NH_2_	0.16 (1.89)	92	47
C_16_-KβAK-NH_2_	Not determined		
C_14_-KKKK-NH_2_	1.22 (18.04)	905	40
C_14_-KKKR-NH_2_	1.01	779	42
C_14_-KRKK-NH_2_	1.25	962	37
C_12_-KKKK-NH_2_	3.10	2216	37
C_12_-KRKK-NH_2_	3.60	2658	40

The CAC values in parentheses were previously determined in unbuffered aqueous solution [37].

**Table 3:** Thermodynamic parameters for the binding of the lipopeptides to POPG and DPPG LUVs (large unilamellar vesicles) and LPS *E. coli* 055:B5.

Peptide		*n*	pK_ITC_	Δ*H* kcal mol^−1^	TΔ*S* kcal mol^−1^	Δ*G* kcal mol^−1^
		POPG				
C_18_-KK-NH_2_		0.91 ± 0.03	5.90 ± 0.22	−0.67 ± 0.03	9.74 ± 0.23	−10.41 ± 0.23
C_16_-KK-NH_2_		1.08 ± 0.04	5.32 ± 0.11	−0.49 ± 0.03	9.14 ± 0.15	−9.62 ± 0.15
C_16_-KGK-NH_2_		0.76 ± 0.05	5.61 ± 0.21	−0.46 ± 0.04	9.56 ± 0.29	−10.02 ± 0.29
C_16_-KβAK-NH_2_		1.54 ± 0.05	5.34 ± 0.11	−0.52 ± 0.02	9.13 ± 0.16	−9.65 ± 0.16
C_16_-KKKK-NH_2_						
	TFA^−^	2.87 ± 0.16	4.73 ± 0.16	−0.37 ± 0.03	8.46 ± 0.22	−8.83 ± 0.21
	AcO^−^	3.70 ± 0.11	5.14 ± 0.15	−0.37 ± 0.01	9.02 ± 0.20	−9.38 ± 0.20
	Cl^−^	6.13 ± 0.32	4.69 ± 0.15	−0.36 ± 0.03	8.41 ± 0.21	−8.76 ± 0.21
C_14_-KKKK-NH_2_		3.94 ± 0.14	6.10 ± 0.42	−0.09 ± 0.01	10.60 ± 0.58	−10.69 ± 0.58
C_14_-KKKR-NH_2_		3.44 ± 0.08	5.97 ± 0.22	−0.14 ± 0.01	10.37 ± 0.30	−10.51 ± 0.30
C_14_-KRKK-NH_2_		4.01 ± 0.07	6.05 ± 0.20	−0.29 ± 0.01	10.34 ± 0.27	−10.63 ± 0.27
		**DPPG**				
C_16_-KKKK-NH_2_						
	TFA^−^	1.51 ± 0.08	5.16 ± 0.16	−1.35 ± 0.10	8.05 ± 0.21	−9.41 ± 0.21
	AcO^−^	1.79 ± 0.05	6.26 ± 0.27	2.30 ± 0.10	13.21 ± 0.38	−10.91 ± 0.37
	Cl^−^	4.36 ± 0.10	4.68 ± 0.10	3.44 ± 0.13	12.20 ± 0.17	−8.76 ± 0.14
C_14_-KKKK-NH_2_		3.39 ± 0.03	5.39 ± 0.06	1.71 ± 0.02	11.44 ± 0.22	−9.72 ± 0.08
C_12_-KKKK-NH_2_		2.61 ± 0.03	5.19 ± 0.04	1.84 ± 0.03	11.29 ± 0.06	−9.45 ± 0.06
		**LPS *E. coli* 055:B5**				
C_16_-KGK-NH_2_		1.67 ± 0.09 6.31 ± 0.26	6.74 ± 0.41 5.48 ± 0.34	2.4 ± 0.36 −1.31 ± 0.15	13.96 ± 0.56 8.54 ± 0.59	−11.56 ± 0.56 −9.84 ± 0.47

**Table 4:** Peptide content and relative peptide content in the samples of C16-KKKK-NH2 with different counterions.

Counterion	Mass Fraction mg/mg	RSD %	Relative Content
No counterion	0.965	2.343	100%
TFA^−^	0.596	5.633	62%
AcO^−^	0.691	5.065	72%
Cl^−^	0.749	5.138	78%

**Table 5:** The average and standard deviation of the APL, bilayer thickness and lateral diffusion coefficients calculated over the last 100 ns of the CG MD simulations.

Model	APL [Å^2^]						Thickness [Å]
	Top Leaflet			Bottom Leaflet			
	RaLPS	POPG	POPE	CDL2	POPG	POPE	
G−	182.03 ± 0.30	-	-	73.54 ± 2.93	64.74 ± 3.17	62.98 ± 0.27	36.60 ± 0.01
G−/peptide	179.83 ± 0.25			74.15 ± 2.32	64.04 ± 2.65	62.13 ± 0.23	36.73 ± 0.09
G+		63.39 ± 0.53	58.58 ± 1.06	-	63.40 ± 0.50	58.53 ± 1.18	39.98 ± 0.02
G+/peptide		34.56 ± 0.77	39.50 ± 0.98		67.63 ± 6.14	63.07 ± 1.31	39.29 ± 0.17
	D [10^−7^ cm^2^/s]						
G−	0.014 ± 0.008			3.94 ± 0.38	5.16 ± 0.17	5.23 ± 0.25	
G−/peptide	0.013 ± 0.006			2.66 ± 0.43	6.46 ± 0.79	4.71 ± 0.03	
G+	-	4.33 ± 0.87	4.68 ± 0.89	-	4.56 ± 0.64	5.01 ± 0.66	
G+/peptide	-	4.21 ± 0.21	4.24 ± 0.09	-	6.55 ± 0.27	5.65 ± 0.81	

## Data Availability

The data may be provided by the corresponding author on request.

## Supplementary Materials

Supplementary materials can be found at https://www.mdpi.com/article/10.3390/antibiotics11111491/s1. Table S1: Characteristics of the lipopeptides, Figure S1: (A) Haemolytic activities of the lipopeptides. (B) Stability of the selected lipopeptides in human serum. (C) Linear dependence between logarithm of the CAC values and the number of carbons in the hydrocarbon chain for compounds with a tetralysine headgroup. (D) Self-diffusion coefficients extracted from PFG NMR diffusion experiments for C_16_-KKKK-NH_2_ and C_16_-RRRR-NH_2_. (E) Far-UV CD spectra of C_16_-KGK-NH_2_ and C_16_-KβAK-NH_2_ in unbuffered and phosphate-buffered solutions. (F) Dependence of the fluorescence intensity of the peptide-thioflavin T complex on the time of incubation, relative to a control (ThT solution in water), Figure S2: Fluorescence images of *S. epidermidis* viability staining. Planktonic cell stained with SYTO9, PI and PI + SYTO9 treated with C_16_-KKKK-NH_2_ up to concentrations of 8, 16, and 32 µg/mL, corresponding to 1× MIC, 2× MIC, and 4×MIC, respectively, negative and positive controls. The bacterial cells with intact membranes are in green, whereas those with damaged membranes are in red., Figure S3: Fluorescence images of *E. coli* viability staining. Planktonic cell stained with SYTO9, PI and PI + SYTO9 treated with C_16_-KKKK-NH_2_ up to concentrations of 16, 32 and 64 µg/mL, corresponding to 1×MIC, 2×MIC, and 4×MIC, respectively, negative and positive controls. The bacterial cells with intact membranes are in green, whereas those with damaged membranes are in red., Figure S4: ITC traces showing heat changes upon titration of 1.3 mM POPG to 0.05 mM peptide solutions at 298.15 K. The bottom curves represent the heat of reaction vs. the POPG:lipopeptide molar ratio, Figure S5: ITC traces showing heat changes upon titration of 1.3 mM POPG to 0.05 and 0.1 mM C_12_-KKKK-NH_2_ (A) and 0.05 and 0.1 mM C_12_-KRKK-NH_2_ (B) at 298.15 K. The bottom curves represent the heat of reaction vs. the POPG:lipopeptide molar ratio, Figure S6: ITC traces showing the heat changes upon titration of 1.3 mM DPPG to 0.05 mM C_16_-KKKK-NH_2_, C_14_-KKKK-NH_2_ and C_14_-KKKK-NH_2_ solutions at 298.15 K. The bottom curves represent the heat of reaction vs. the DPPG:lipopeptide molar ratio, Figure S7: ITC traces showing the heat changes upon titration of the 1.3 mM POPG to 0.05 mM C_16_-KKKK-NH_2_ solutions at 298.15 K with increasing concentration of trifluoroacetate ions (TFA^−^) in the sample (peptide-sodium trifluoroacetate molar ratios of 1:0, 4:1, 2:1 and 1:1). The bottom curves represent the heat of reaction, measured by peak integration, vs. the POPG:lipopeptide molar ratio.

## Abbreviations

CAC	Critical aggregation concentration
C_18_	Stearic acid
C_16_	Palmitic acid
C_14_	Myristic acid
C_12_	Lauric acid
CDL2	Cardiolipin 2
CG MD	Coarse-grained molecular dynamics
DPPC	1,2-Dipalmitoyl-*sn*-glycero-3-phosphocholine
DPPG	1,2-Dipalmitoyl-*sn*-glycero-3-[phospho-rac-(1-glycerol)]
LUV	Large unilamellar vesicle
MLV	Multilayer vesicle
POPC	1-Palmitoyl-2-oleoyl-*sn*-glycero-3-phosphocholine
POPE	1-Palmitoyl-2-oleoyl-*sn*-glycero-3-phosphatidylethanolamine
POPG	1-Palmitoyl-2-oleoyl-*sn*-glycero-3-[phospho-rac-(1-glycerol)]
RaLPS	Ra mutant rough chemotype lipopolysaccharide
