# Supplementary material for: Understanding the Role of Self-Assembly and Interaction with Biological Membranes of Short Cationic Lipopeptides in the Effective Design of New Antibiotics
Source: Antibiotics (Basel). 2022 Oct 27;11(11):1491. doi: 10.3390/antibiotics11111491 (PMC9686977; doi:10.3390/antibiotics11111491)
Supplement: Supplementary file 1 [file antibiotics-11-01491-s001.zip › antibiotics-1995346-supplementary.pdf]

Supplementary Materials

Understanding the role of self-assembly and interaction with biological membranes of short cationic lipopeptides in the effective design of new antibiotics

Oktawian Stachurski[1], Damian Neubauer[2], Aleksandra Walewska[1], Emilia Iłowska[1], Marta Bauer[2], Sylwia Bartoszewska[2], Karol Sikora[2], Aleksandra Hać[3], Dariusz Wyrzykowski[1], Adam Prahl[1], Wojciech Kamysz[2], Emilia Sikorska[1*]

[1] Faculty of Chemistry, University of Gdansk, Wita Stwosza 63, 80-308 Gdansk, Poland
[2] Faculty of Pharmacy, Medicinal University of Gdansk, Al. Gen. J. Hallera 107, 80-416 Gdansk, Poland
[3] Faculty of Biology, University of Gdansk, Wita Stwosza 59, 80-308 Gdansk, Poland

*Correspondence: emilia.sikorska@ug.edu.pl

Keywords: antimicrobial peptides; lipopeptides; peptide–membrane interactions; self–assembly.

Table S1. Characteristics of the studied lipopeptides

Compound	t'_R [min]	Average mass [Da]	MS analysis		
			z	m/z calc.	m/z found
C_{18}-KK-NH_2	12.22	539.85	1	540.48	540.58
			2	270.75	271.06
			Ion $[2M+H]^+$	1079.96	1079.87
C_{16}-KK-NH_2	10.18	511.79	1	512.45	512.70
			2	256.73	257.02
C_{16}-KβAK-NH_2	10.28	582.87	1	583.49	583.63
			2	292.25	292.63
C_{16}-KGK-NH_2	10.08	568.84	1	569.48	569.67
			2	285.24	285.60
C_{16}-KKKK-NH_2	8.51	768.14	1	768.64	768.80
			2	384.83	385.23
			3	256.89	257.29
			4	192.92	-
C_{14}-KKKK-NH_2	6.86	740.09	1	740.61	740.76
			2	370.81	371.10
			3	247.54	247.80
			4	186.91	-
C_{14}-KRKK-NH_2	7.05	768.10	1	768.62	768.78
			2	384.81	385.27
			3	256.88	257.32
			4	192.91	-
C_{14}-KKKR-NH_2	6.92	768.10	1	768.62	768.64
			2	384.81	385.15
			3	256.88	257.27
			4	192.91	193.27
C_{12}-KKKK-NH_2	5.09	712.03	1	712.58	712.72
			2	356.79	357.11
			3	238.20	238.59
			4	178.90	179.33
C_{12}-KRKK-NH_2	5.24	740.05	1	740.59	740.75
			2	370.80	371.20
			3	247.53	247.95
			4	185.90	-

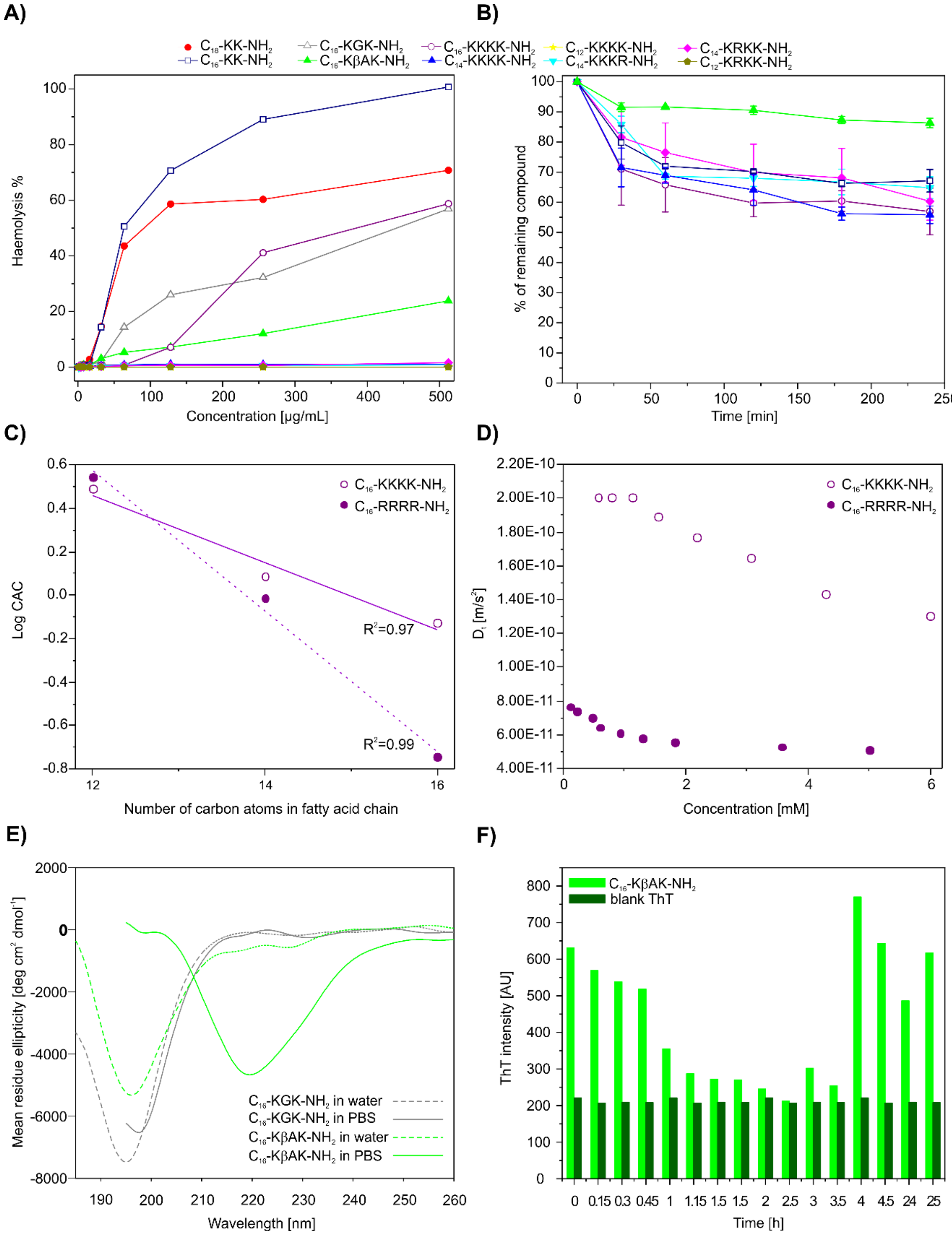

Figure S1. (**A**) Haemolytic activities of the lipopeptides. (**B**) Stability of the selected lipopeptides in human serum. (**C**) Linear dependence between logarithm of the CAC values and the number of carbons in the hydrocarbon chain for compounds with a tetralysine headgroup. (**D**) Self-diffusion coefficients extracted from PFG NMR diffusion experiments for C_{16}-KKKK-NH_2 and C_{16}-RRRR-NH_2. (**E**) Far-UV CD spectra of C_{16}-KGK-NH_2 and C_{16}-KβAK-NH_2 in unbuffered and phosphate-buffered solutions. (**F**) Dependence of the fluorescence intensity of the peptide-thioflavin T complex on the time of incubation, relative to a control (ThT solution in water).

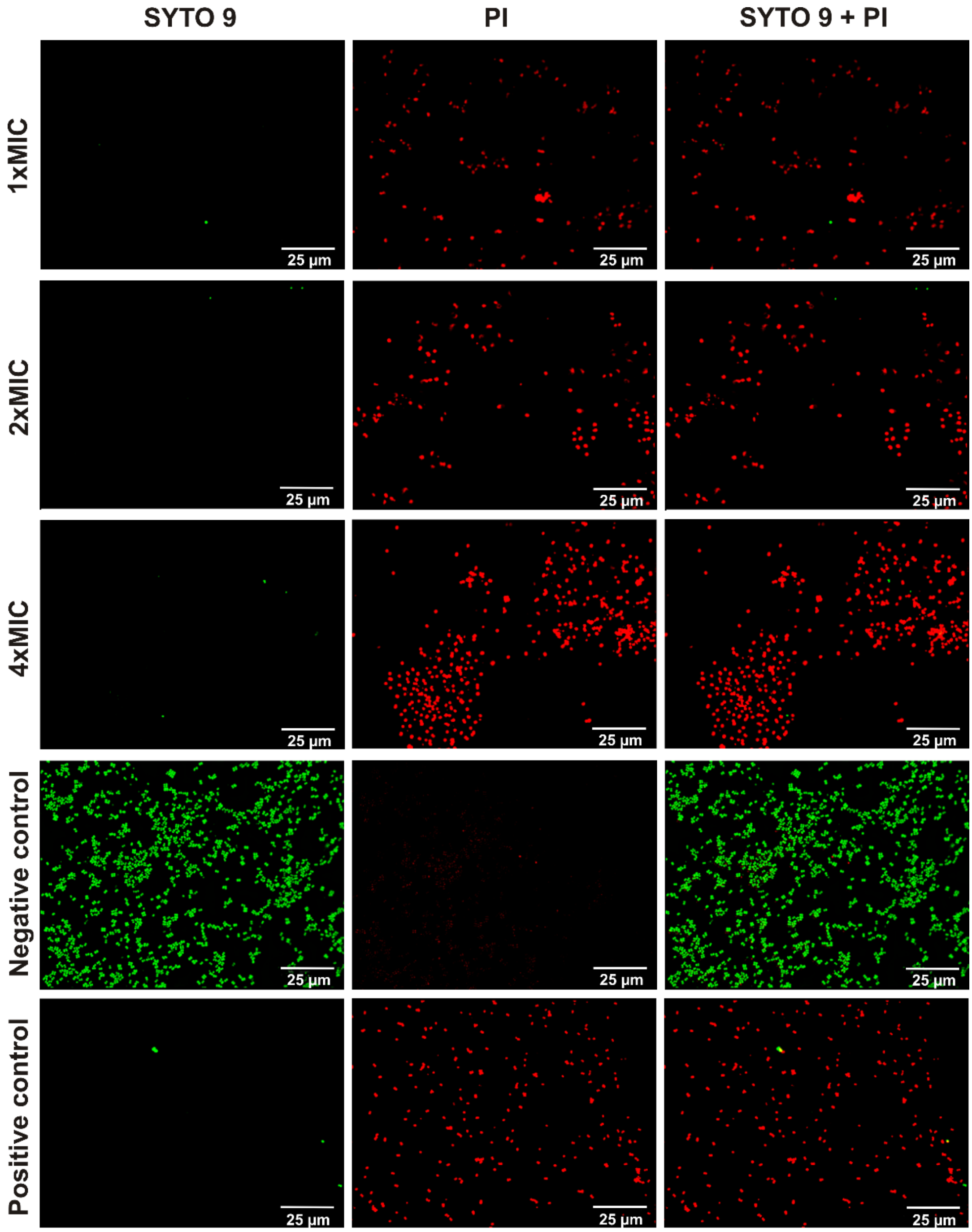

Figure S2. Fluorescence images of *S. epidermidis* viability staining. Planktonic cell stained with SYTO9, PI and PI + SYTO9 treated with C_{16}-KKKK-NH_2 up to concentrations of 8, 16, and 32 µg/mL, corresponding to 1×MIC, 2×MIC, and 4×MIC, respectively, negative and positive controls. The bacterial cells with intact membranes are in green, whereas those with damaged membranes are in red.

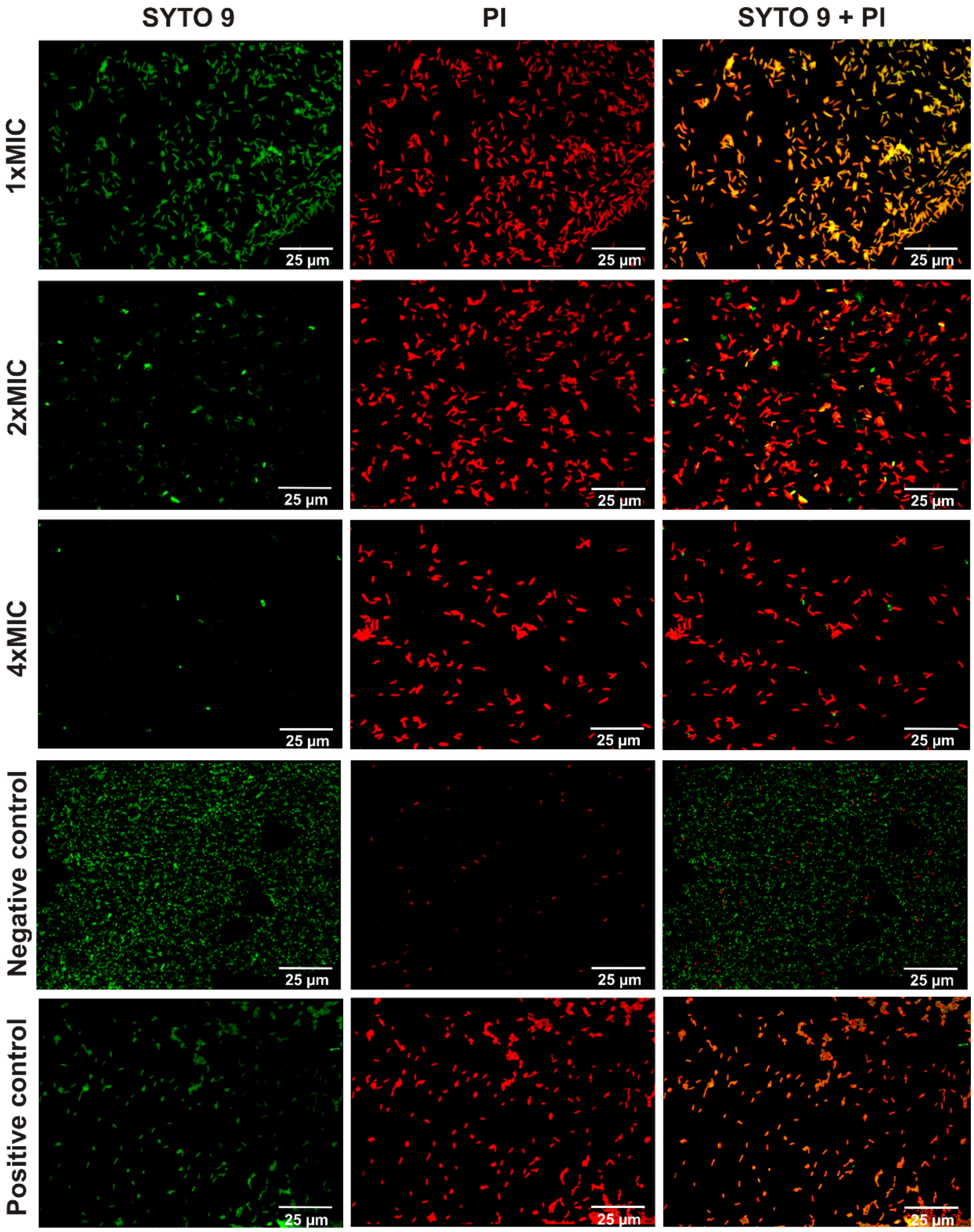

Figure S3. Fluorescence images of *E. coli* viability staining. Planktonic cell stained with SYTO9, PI and PI + SYTO9 treated with C_{16}-KKKK-NH_2 up to concentrations of 16, 32 and 64 µg/mL, corresponding to 1×MIC, 2×MIC, and 4×MIC, respectively, negative and positive controls. The bacterial cells with intact membranes are in green, whereas those with damaged membranes are in red.

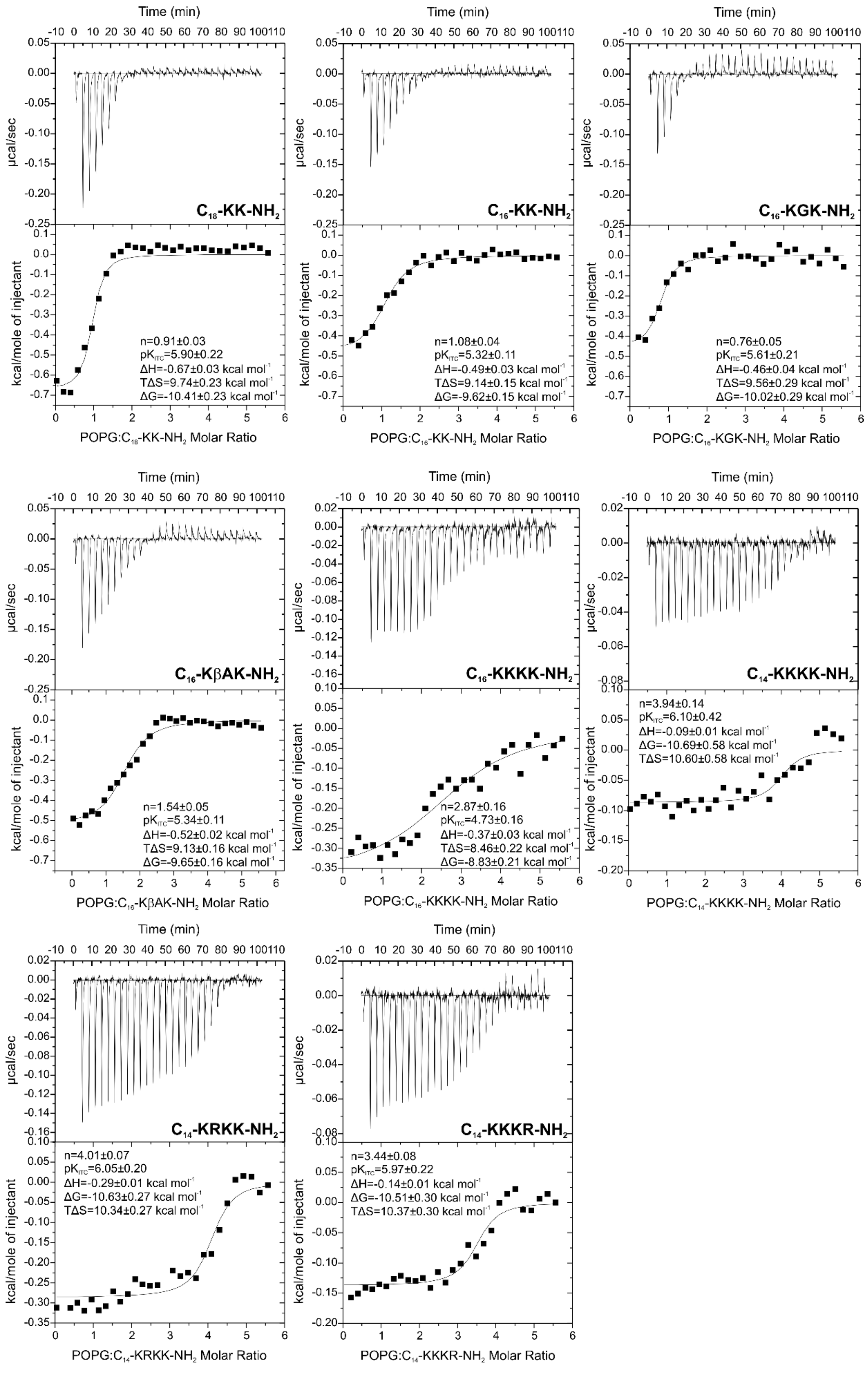

Figure S4. ITC traces showing heat changes upon titration of 1.3 mM POPG to 0.05 mM peptide solutions at 298.15 K. The bottom curves represent the heat of reaction *vs.* the POPG:lipopeptide molar ratio.

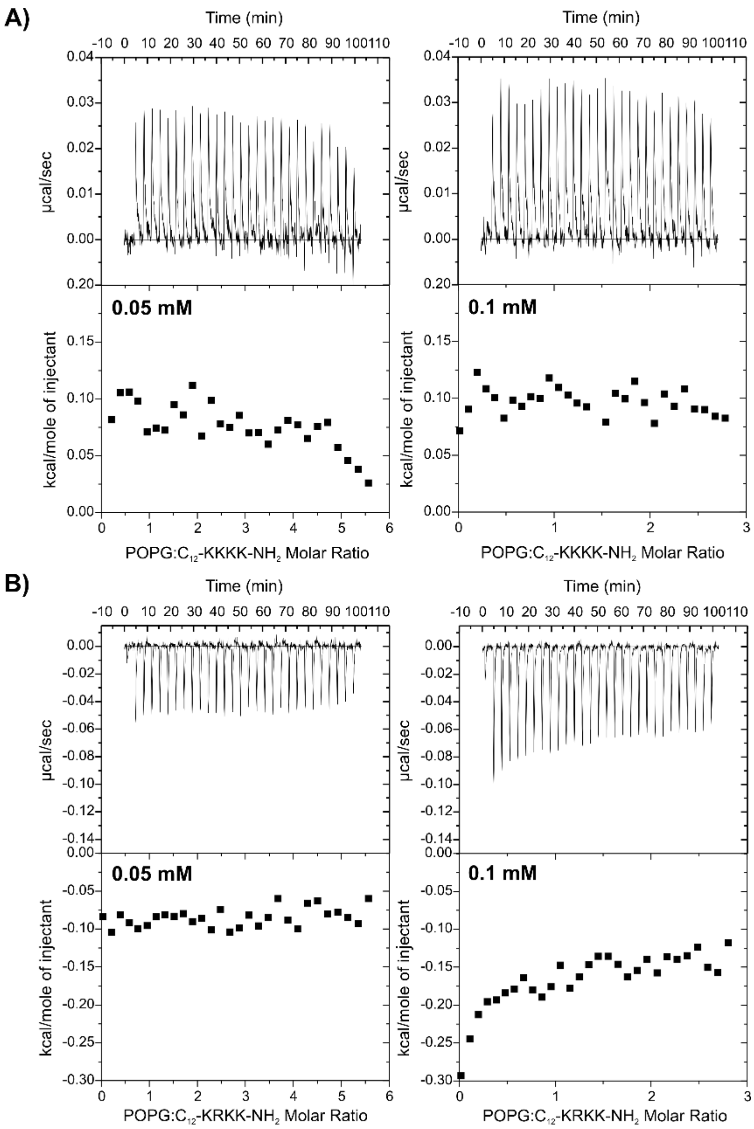

Figure S5. ITC traces showing heat changes upon titration of 1.3 mM POPG to 0.05 and 0.1 mM C_{12}-KKKK-NH_2 (**A**) and 0.05 and 0.1 mM C_{12}-KRKK-NH_2 (**B**) at 298.15 K. The bottom curves represent the heat of reaction *vs.* the POPG:lipopeptide molar ratio.

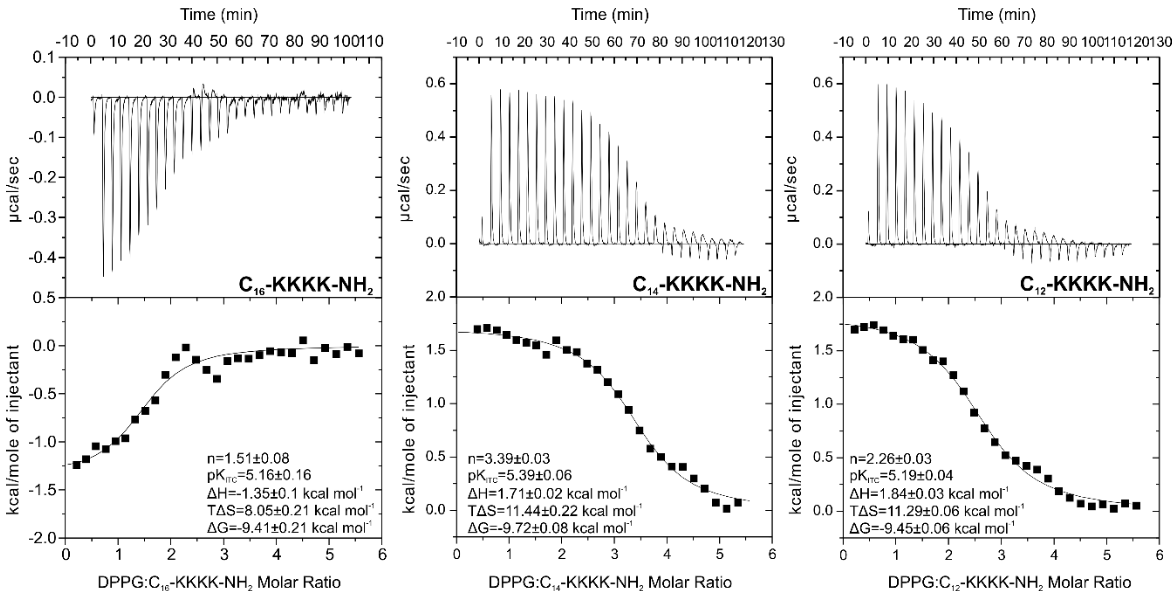

Figure S6. ITC traces showing the heat changes upon titration of 1.3 mM DPPG to 0.05 mM C_{16}-KKKK-NH$_2$, C_{14}-KKKK-NH$_2$ and C_{14}-KKKK-NH$_2$ solutions at 298.15 K. The bottom curves represent the heat of reaction *vs.* the DPPG:lipopeptide molar ratio.

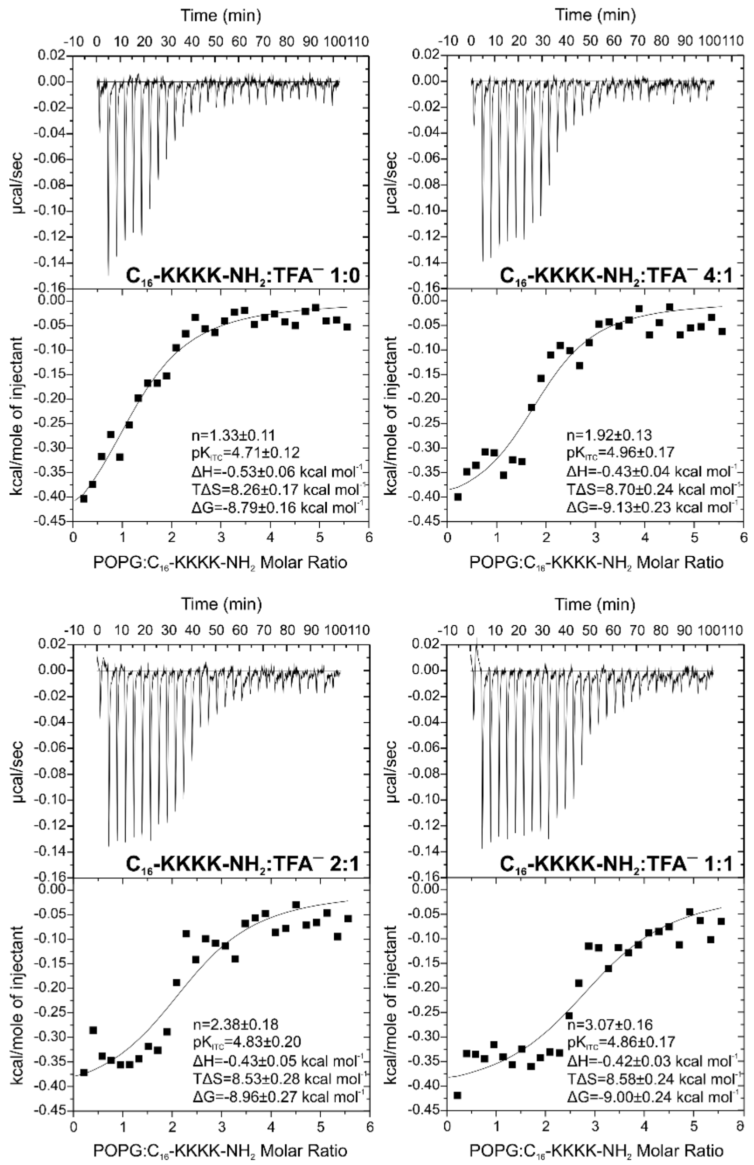

Figure S7. ITC traces showing the heat changes upon titration of the 1.3 mM POPG to 0.05 mM C_{16}-KKKK-NH_2 solutions at 298.15 K with increasing concentration of trifluoroacetate ions (TFA^-) in the sample (peptide-sodium trifluoroacetate molar ratios of 1:0, 4:1, 2:1 and 1:1). The bottom curves represent the heat of reaction *vs.* the POPG:lipopeptide molar ratio.
